# Artificial Intelligence Revolution in Transcriptomics: From Single Cells to Spatial Atlases

**DOI:** 10.1002/advs.202518949

**Published:** 2025-12-12

**Authors:** Shixin Li, Tianxiang Xiao, Yuanyuan Lan, Chengxiao Wu, Zhouying Li, Rong Liu, Qing Fang, Chao Zhang

**Affiliations:** ^1^ State Key Laboratory of Genetic Evolution & Animal Models, Kunming Institute of Zoology Chinese Academy of Sciences Kunming 650201 China; ^2^ Yunnan Key Laboratory of Biodiversity Information, Kunming Institute of Zoology Chinese Academy of Sciences Kunming 650201 China; ^3^ National Research Facility for Phenotypic and Genetic Analysis of Model Animals (Primate Facility), Kunming Institute of Zoology Chinese Academy of Sciences Kunming Yunnan 650107 China; ^4^ University of Chinese Academy of Sciences Beijing 100049 China; ^5^ School of Life Sciences Peking University Beijing 100871 China; ^6^ Center for Bioinformatics Peking University Beijing 100871 China

**Keywords:** agent, artificial intelligence, deep learning, foundation model, review, single cell RNA‐seq, spatial transcriptomics

## Abstract

Single‐cell RNA sequencing (scRNA‐seq) and spatial transcriptomics (ST) have revolutionized the study of cellular heterogeneity and tissue organization. However, the increasing scale and complexity of these data demand more powerful and integrative computational strategies. Although conventional statistical and machine learning methods remain effective in specific contexts, they face limitations in scalability, multimodal integration, and generalization. In response, artificial intelligence (AI) has emerged as a transformative force, enabling new modes of analysis and interpretation. In this review, we survey AI applications across the transcriptomic analysis workflow—from initial preprocessing through key downstream analyses such as trajectory inference, gene regulatory network reconstruction, and spatial domain detection. For each analytical task, we trace the developmental trajectory and evolving trends of AI models, summarize their advantages, limitations, and domain‐specific applicability. We also highlight key innovations, ongoing challenges, and future directions. Furthermore, this review provides practical guidance to assist researchers in model selection and support developers in the design of novel AI tools. An online companion supplement providing an in‐depth look at all methods discussed: https://zhanglab‐kiz.github.io/review‐ai‐transcriptomics.

## Graphical abstract

Single‐cell RNA sequencing and spatial transcriptomics have unveiled cellular heterogeneity and tissue organization with unprecedented resolution. Artificial intelligence (AI) now plays a pivotal role in interpreting these complex data. This review systematically surveys AI applications across the entire analytic workflow and offers practical guidance to assist researchers in model selection and support developers in designing novel AI tools.

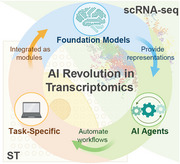

## 1 Introduction

The advent of single‐cell RNA sequencing (scRNA‐seq) has marked a profound paradigm shift in biological research, enabling the dissection of the intricate transcriptional landscapes at cellular resolution.^[^ ^1^, ^2^ ^]^ By profiling transcriptomes across thousands to millions of individual cells, this technology provides unprecedented resolution for investigating cellular heterogeneity.^[^ ^3^, ^4^, ^5^ ^]^ This capability enables the discovery of rare or novel cell populations and reveals cellular and molecular mechanisms underlying complex biological processes, from embryogenesis to the pathogenesis of diseases such as cancer and neurodegeneration.^[^ ^6^, ^7^, ^8^ ^]^

While scRNA‐seq excels at defining cell types and uncovering heterogeneity, it requires tissue dissociation, which results in irreversible loss of spatial context.^[^ ^9^ ^]^ To overcome this limitation, Spatial Transcriptomics (ST) has emerged as a revolutionary complementary technology that maps transcriptomes within intact tissue sections.^[^ ^10^, ^11^ ^]^ This preservation of spatial context is biologically crucial, as cells function not in isolation but through precise spatial organization and interactions within tissue microenvironments. ST thus enables a holistic understanding of tissue architecture, cellular neighborhoods, and cell‐cell communication,^[^ ^8^, ^9^, ^12^ ^]^ bridging the gap between cellular identity and tissue function to reveal how spatial organization governs gene expression and underlies disease pathogenesis.

However, scRNA‐seq and ST data present significant analytical challenges stemming from several inherent complexities. 1) high dimension, where profiling thousands of genes per cell creates vast feature spaces in which biological signals are obscured by noise;^[^ ^13^, ^14^ ^]^ 2) data sparsity, where abundant zero counts (“dropouts”) arising from both true biological absence and technical artifacts mask expression dynamics of low‐abundance genes;^[^ ^15^, ^16^ ^]^ 3) systematic technical noise and batch effects that confounding biological comparisons;^[^ ^13^, ^17^ ^]^ and 4) ST‐specific challenges including cell deconvolution and integration with high‐resolution imaging data.^[^ ^18^ ^]^ Collectively, these characteristics render traditional bioinformatics tools inadequate, necessitating more advanced computational methods to reliably extract meaningful insights.

Artificial intelligence (AI), particularly deep learning, offers powerful strategies for tackling the growing analytical challenges in biology and is transforming the field.^[^ ^19^, ^20^ ^]^ Traditional statistical and probabilistic approaches, in contrast, struggle with the “curse of dimensionality” and restrictive modeling assumptions, making it difficult to capture the complexity of high‐dimensional, noisy biological data. Deep learning overcomes these barriers by learning effective representations and complex, non‐linear relationships directly from high‐dimensional data, thereby bypassing the restrictive modeling assumptions and manual feature engineering that limit traditional approaches. Moreover, it enables end‐to‐end learning to integrate heterogeneous, multimodal datasets into unified representations, thereby improving data utilization and yielding more comprehensive insights.^[^ ^21^, ^22^, ^23^ ^]^ Critically, as biological technologies generate increasingly large and diverse datasets, deep learning leverages this scale to develop more robust, generalizable models. This synergy accelerates the development of advanced architectures whose predictive and generative capabilities expand biological discovery and drive innovation in experimental design and technology development.

**Figure 1:**
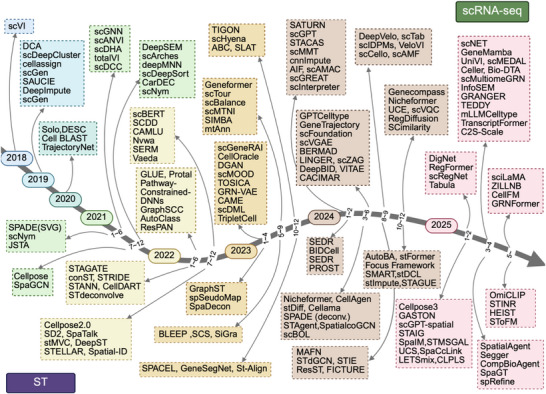
Timeline of AI‐based models for scRNA‐seq and ST data analysis. This timeline depicts the development of AI‐based models, with scRNA‐seq models shown above the line and ST models shown below.

Herein, we review recent advances in AI models for scRNA‐seq and ST data analysis. These models address key challenges across the analytical spectrum, from data processing (denoising, imputation, batch correction) to biological discovery (cell type annotation, trajectory inference, gene regulatory network reconstruction) (**Figure** **1**). We systematically examine the principles, characteristics, and implementations of state‐of‐the‐art approaches, summarizing their respective advantages, limitations, and domain‐specific applications. Finally, we discuss future challenges and opportunities for AI‐driven scRNA‐seq and ST data analysis.

## 2 Architecture of AI Models for Single‐cell and Spatial Transcriptomics

**Figure 2:**
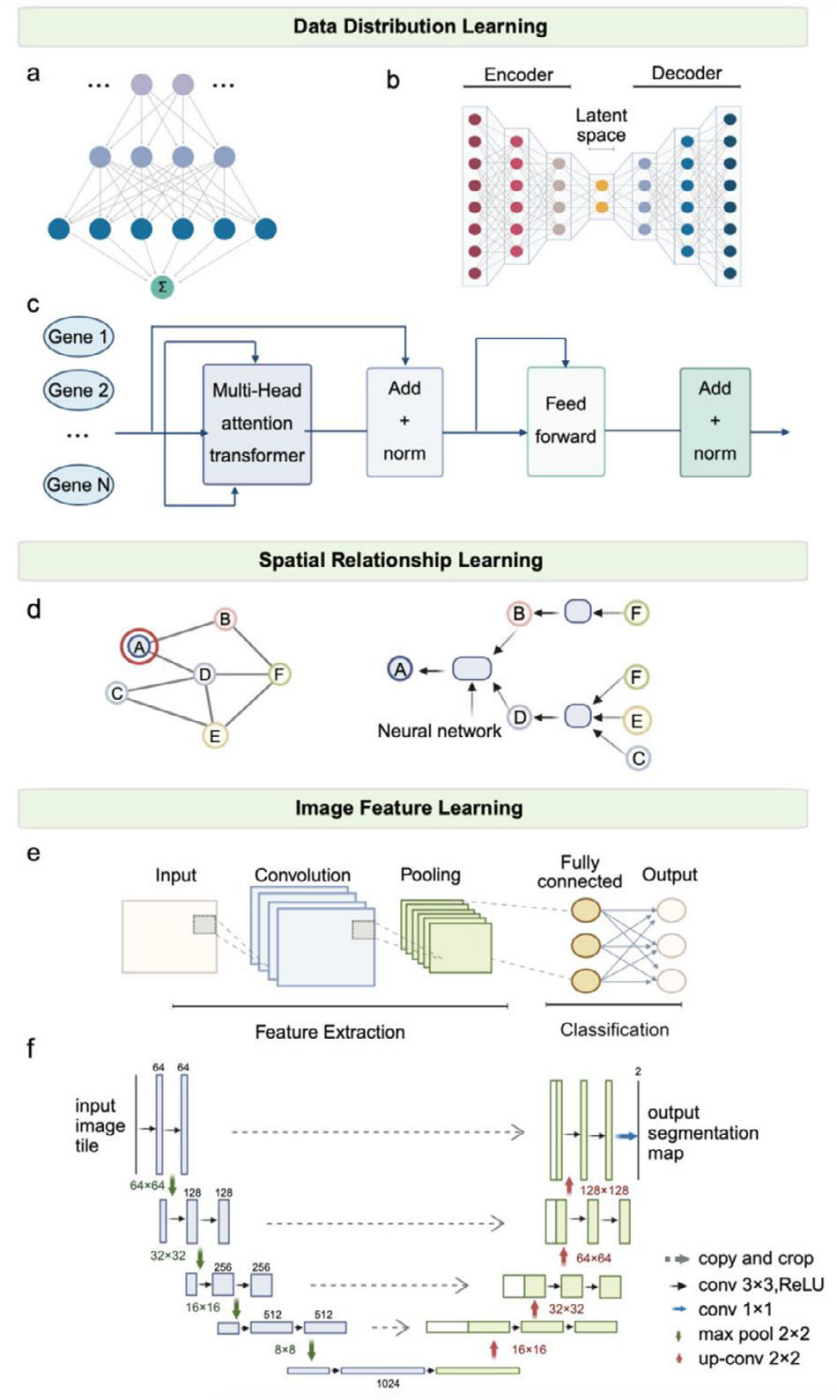
Architecture of AI models for single‐cell and spatial transcriptomics. The models are organized into three main categories: a) models for data distribution learning (e.g., autoencoders, transformers), b) models for spatial relationship learning (e.g., graph neural networks), and c) models for image feature learning (e.g., convolutional neural networks, U‐net).

The pace of AI advancement in both academia and industry has far outstripped that of many other disciplines,^[^ ^24^ ^]^ generating diverse model architectures for single‐cell and spatial transcriptomics. This review focuses on AI models that have exerted substantial influence, achieved widespread adoption, or established methodological foundations in the field. For clarity, we classify these architectures into three primary categories (**Figure** **2**).

### 2.1 Models for Data Distribution Learning

The fully connected (FC) layer, as a fundamental component of modern deep learning architectures, consists of a set of “neurons” that mimic the functional organization of the biological brain (Figure 2a).^[^ ^25^ ^]^ Each neuron computes a weighted sum of its input signals followed by a nonlinear activation function (e.g., ReLU), thereby enhancing the model's capacity to capture complex relationships.^[^ ^26^ ^]^ Beyond this basic computational, the FC layer also serves several critical functions in more complex models. Specifically, it can reshape information by reducing or expanding dimensions to facilitate pattern learning.^[^ ^27^, ^28^ ^]^ Moreover, it frequently operates as a classification head at pipeline's final stage or as an internal module for encoding and decoding data.^[^ ^29^ ^]^

The variational autoencoder (VAE) is one of the most widely used architectures for modeling scRNA‐seq data distributions (Figure 2b). It consists of an encoder and a decoder, both typically implemented with multiple fully connected layers (i.e., multilayer perceptrons, MLPs). The encoder estimates parameters such as mean and variance, mapping input data into a latent space that typically follows a Gaussian prior. The decoder then reconstructs the original data from this latent representation using a specified probabilistic model.^[^ ^29^ ^]^ In single‐cell transcriptomics, negative binomial (NB) and zero‐inflated negative binomial (ZINB) distributions are commonly adopted at this step to capture over‐dispersed and zero‐inflated in count‐based scRNA‐seq data. NB extends the Poisson model via a dispersion parameter to accommodate biological variability, while ZINB further incorporating a mixture component to explicitly distinguish true biological zeros from technical dropout arising from low RNA capture efficiency.^[^ ^30^ ^]^ Leveraging this powerful and biologically plausible reconstruction capacity, the VAEs effectively capture single‐cell data distributions and have been extensively applied to dimension reduction, batch effect correction, denoising, and imputation.^[^ ^21^, ^22^, ^23^ ^]^

Another seminal architecture, the Transformer (Figure 2c), serves as the foundation for modern large language models (LLMs) such as DeepSeek, ChatGPT,^[^ ^31^ ^]^ and increasingly for Foundation Models (FMs) in scRNA‐seq and ST. Its key innovation—the multi‐head attention mechanism, enables simultaneous capture of global and local dependencies across long‐range sequences. In single‐cell analysis, this architecture naturally treat each cell as a “document” and expressed genes as tokens, analogous to natural language processing (NLP).^[^ ^23^, ^32^, ^33^, ^34^ ^]^ Gene expression vectors are transformed within the attention mechanism into query, key, and value representations, where attention scores derived from query‐key similarities govern information sharing between genes. This mechanism provides an interpretable framework for modeling gene‐gene interactions, enabling applications such as perturbation analysis, drug response prediction, and virtual gene knockout experiments.^[^ ^35^, ^36^ ^]^ Beyond gene expression, the Transformer's flexible tokenization can incorporate metadata—including cell type labels, batch identifiers, and spatial coordinates—as auxiliary tokens. These tokens integrate contextual information with gene expression profiles, supporting tasks such as batch effect correction, biological context learning, and multimodal data integrations.^[^ ^34^, ^35^, ^37^, ^38^ ^]^

### 2.2 Models for Spatial Relationship Learning

A fundamental characteristic of biological data is its inherent spatial structure. These spatial relationships can be represented as networks, such as protein‐protein interactions (PPI), cell signaling pathways, gene regulatory network (GRN), and cell‐cell interactions (CCI). Graph neural networks (GNNs), a landmark architecture, are routinely employed to model such non‐Euclidean structures (Figure 2d).^[^ ^39^ ^]^ Accordingly, applying GNNs to capture these networks in single‐cell data, as well as the explicit spatial information in spatial transcriptomics, is both intuitive and effective.^[^ ^40^ ^]^

GNNs are generally composed of nodes and edges. Nodes represent biological entities (e.g., cells in scRNA‐seq or spots or segmented cells in ST), while edges encode relationships such as transcriptional similarity, spatial proximity, or prior knowledge like PPI. Each node is associated with an initial feature vector, typically its gene expression profile, which may be concatenated with additional information such as image‐derived features.^[^ ^41^, ^42^, ^43^ ^]^ As illustrated in Figure 2d, node updates are determined by aggregating information from neighbors through operations such as summation, mean, max pooling, or attention. This neighbor aggregation mechanism enables GNNs to captured complex dependencies, demonstrating strong performance in both scRNA‐seq and ST data. In scRNA‐seq, constructing a cell‐cell similarity graph enables methods such as scGAE^[^ ^44^ ^]^ to improve trajectory inference and cluster separation, while scGNN^[^ ^22^ ^]^ and scVGAE^[^ ^45^ ^]^ leverage GNN‐based architectures for effective imputation. For ST data, GNNs similarly excel by exploiting spatial organization. For instance, SpaGCN^[^ ^42^ ^]^ categorizes tissues by integrating gene expression with spatial location, thereby delineating functionally consistent domains. Likewise, Spatalk^[^ ^46^ ^]^ incorporates extensive prior biological knowledge through a knowledge graph modeling ligand‐receptor downstream signaling pathways, supporting both single‐cell and spot‐based ST data.

### 2.3 Models for Image Feature Learning

While the conventional single‐cell analysis primarily uses matrix‐based data, modern biotechnology increasingly provides rich multimodal information. In ST, Hematoxylin and Eosin (H&E)‐stained tissue images are paired with their corresponding expression matrices, motivating computational methods that extract image features and integrate them with transcriptional data, leveraging morphological information as a biological prior to ensure the resulting models are histologically grounded and biologically interpretable. For this purpose, convolutional neural networks (CNNs) are particularly well‐suited(Figure 2e).^[^ ^47^ ^]^ As a fundamental architecture for image processing, CNNs have become the most prominent and widely adopted approach for image feature extraction in deep learning.

Although CNNs are effective for extracting image‐level features, segmentation tasks—particularly cell segmentation—require specialized architectures engineered for accurate pixel‐level prediction. A prominent example is U‐Net,^[^ ^48^ ^]^ (Figure 2f). As shown in Figure 2f, U‐Net adopts a symmetrical architecture with two branches: an encoder for downsampling and a decoder for upsampling. Skip connections link high‐resolution features from the encoder with the corresponding upsampled features in the decoder, enabling accurate pixel‐level segmentation. Despite its structural simplicity, U‐Net performs exceptionally well in segmentation tasks. For ST data, U‐Net‐based models exhibit robust performance across diverse tissue types and imaging conditions, establishing them a reliable choice for segmentation applications.^[^ ^49^, ^50^, ^51^, ^52^ ^]^

## 3 The Application of AI in scRNA‐seq

While scRNA‐seq has revolutionized biological research, its high‐dimensional and sparse data, together with technical noise, pose significant analytical challenges. AI has emerged as a powerful solution, delivering transformative impacts and novel insights across the entire spectrum of scRNA‐seq analysis.

**Figure 3:**
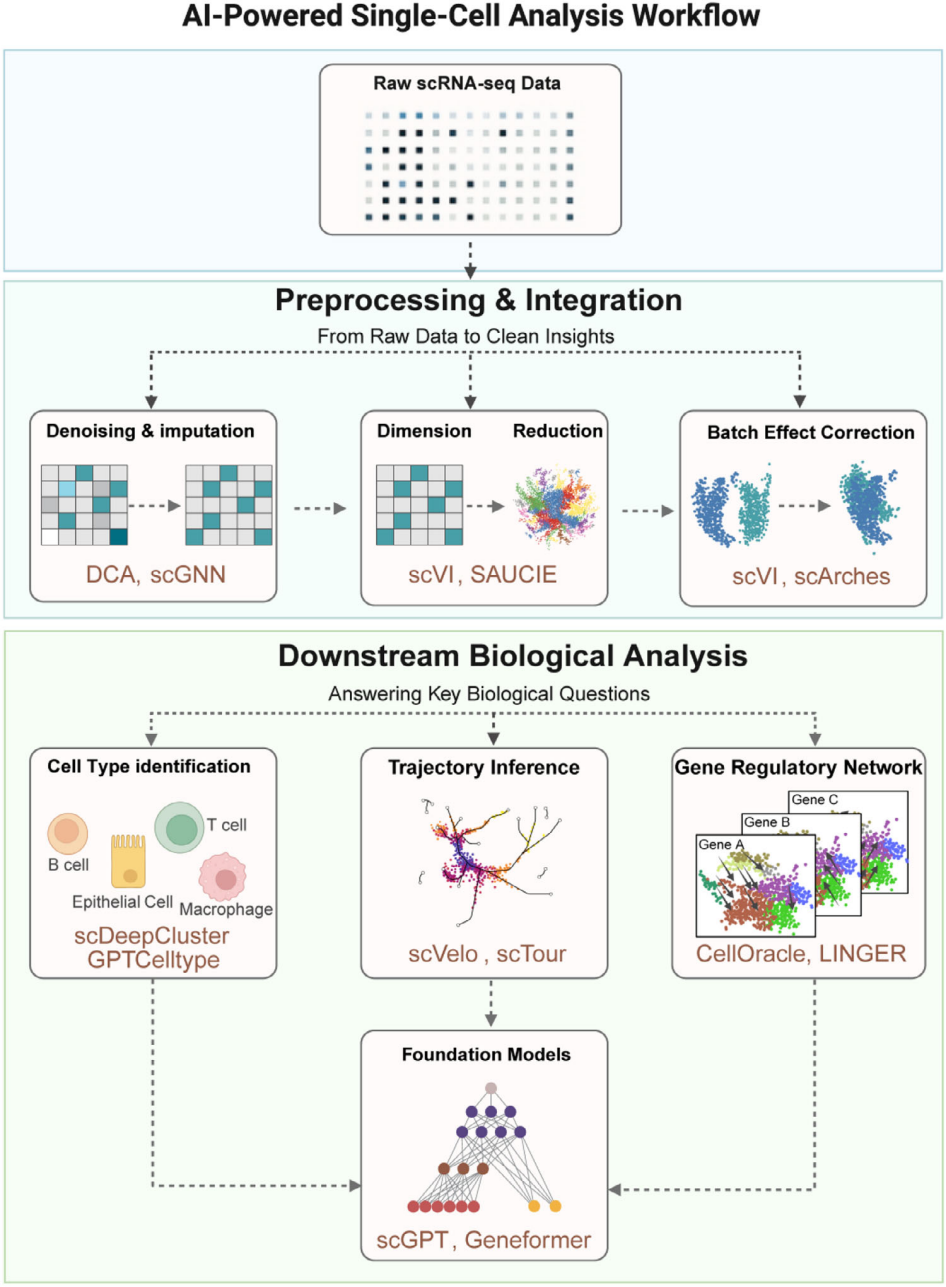
AI‐powered single‐cell analysis workflow. The pipeline consists of two main stages: 1) Preprocessing and integration, including denoising and imputation, dimension reduction, and batch effect correction to generate harmonized data representations; 2) Downstream biological analysis, encompassing cell identification, trajectory inference, and gene regulatory network reconstruction, with Foundation Models providing transferable representations across tasks.

The analytical journey in single‐cell genomics typically follows a structured workflow, progressing from raw data to biological insight (**Figure** **3**). The first stage, preprocessing and integration, is essential for curating and refining raw signals. In this stage, AI models play an increasingly important role, offering sophisticated methods to learn and correct for technical noise, data sparsity (imputation), and experimental artifacts (batch effects). This process effectively harmonizes the data and distills the high‐dimensional gene space into robust, biologically informative low‐dimensional representations. Once the data are harmonized, the focus shifts to downstream biological analysis, which addresses core questions including the identification of novel and known cell types through clustering and annotation, the reconstruction of developmental trajectories to capture dynamic processes, and the inference of GRNs that govern cellular identity. Beyond task‐specific approaches, FMs have recently emerged as versatile frameworks capable of supporting multiple analytical tasks simultaneously.

Accordingly, we structure our review of AI methods for scRNA‐seq around this workflow.

### 3.1 AI for scRNA‐seq Data Preprocessing

AI‐based approaches have demonstrated high effectiveness in essential preprocessing tasks for single‐cell RNA sequencing, including dimension reduction, denoising of expression matrices, imputation of missing values, and detection of artifacts such as doublets. By transforming raw signals into tractable and biologically meaningful representations, these methods establish the foundation for reliable downstream analyses.^[^ ^13^ ^]^

#### 3.1.1 Denoising and Imputation

Noise in scRNA‐seq is diverse and can generally be attributed to biological and technical factors. Biological noise arises from cell state heterogeneity and the stochastic burst‐like nature of gene expression, whereas technical noise—including variability in capture efficiency and differences in sequencing depth—obscures true biological signals and contributes to artifacts such as dropout events, thereby compromising downstream analysis reliability.^[^ ^15^ ^]^ To overcome these limitations, AI‐based models learn the underlying data distributions and relational contexts to enable faithful recovery of missing values while preserving biological heterogeneity. This challenge has driven the field's evolution from early deep learning frameworks^[^ ^22^, ^45^, ^53^, ^54^, ^55^, ^56^, ^57^, ^58^, ^59^, ^60^ ^]^ to advanced generative models^[^ ^61^, ^62^, ^63^, ^64^, ^65^ ^]^ specifically tailored for single‐cell data.

The evolution of AI tools begins with basic deep learning models that independently model the statistical properties of each cell.^[^ ^53^, ^54^, ^55^, ^56^, ^57^ ^]^ Deep autoencoders emerge as a highly influential generative architecture in these early stages, though their reconstruction efficacy critically depends on selecting appropriate loss functions. For instance, the seminal DCA implementation^[^ ^54^ ^]^ employs a ZINB loss function that disentangles true biological zeros from technical dropouts, preserving gene‐gene dependencies to reconstruct developmental trajectories and uncover subtle cellular phenotypes. Other frequently employed losses include Mean Squared Error (MSE), which offers generality with fewer distributional restrictions but may lack specificity, and NB loss, which is more suitable than ZINB for scenarios with relatively lower dropout rates.

A subsequent wave of models shift from modeling cells in isolation to explicitly leveraging intercellular relationships using GNNs.^[^ ^22^, ^45^, ^58^, ^59^, ^60^ ^]^ The core insight is that cell‐cell similarities provide a powerful inductive bias for denoising: a gene's expression in one cell can be informed by its expression in highly similar neighbors. The influential scGNN method^[^ ^22^ ^]^ leverages this principle by constructing graphs from regulatory signals of highly variable genes, ensuring the resulting topology accurately reflects true cellular similarity. This graph structure, when processed by a graph convolutional network (GCN), implements a dual‐guidance mechanism—combining global topology with local similarity—that effectively eliminates noise while reliable inferring single‐cell expression matrices.

Although graph‐based models have proven effective, contemporary research increasingly explores more advanced generative models.^[^ ^61^, ^62^, ^63^, ^64^, ^65^ ^]^ Diffusion models, exemplified by scIDPMs,^[^ ^65^ ^]^ now represent the state‐of‐the‐art, achieving the lowest Mean Absolute Error (MAE) (e.g., 11.001) in imputation tasks and improving downstream cell clustering to near‐perfect scores (e.g., ARI≈0.98; NMI≈0.98). These models learn data distributions by denoising artificially corrupted samples, typically generated by adding Gaussian noise (zero mean, unit variance). However, such Gaussian perturbations differ substantially from the discrete, over‐dispersed, and zero‐inflated distributions characteristic of scRNA‐seq data. This mismatch suggests that future work should focus on noise addition strategies tailored to the statistical properties of single‐cell data, potentially improving generative modeling fidelity.

#### 3.1.2 Dimension Reduction

scRNA‐seq data are inherently high‐dimensional, with complex nonlinear correlations embedded in cellular states. Conventional dimension reduction (DR) methods, such as PCA, struggle to capture these nonlinear structures. In contrast, deep learning approaches have shown clear advantages by learning more expressive, low‐dimensional representations that better preserve the underlying biological variability.^[^ ^14^ ^]^ Autoencoder‐based approaches exploited their capacity to model data distributions for effective DR of scRNA‐seq data.^[^ ^21^, ^44^, ^66^, ^67^ ^]^ Recently, new methods introduce biologically meaningful constraints and prior knowledge, enhancing DR performance as well as the interpretability and biological soundness of the learned representations.^[^ ^68^, ^69^, ^70^, ^71^, ^72^ ^]^

Autoencoders (AEs) are frequently applied in the development of DR tools for scRNA‐seq data due to its direct and efficient nature. A major milestone in this line of work is scVI,^[^ ^21^ ^]^ which successfully decouples biological variability from technical artifacts, such as batch effects and library size, and has since become a cornerstone of the widely used scvi‐tools ecosystem. SAUCIE,^[^ ^67^ ^]^ another advanced VAE‐based method, integrates regularization across its layers to unify noise reduction, imputation, batch correction, and clustering‐based visualization within a single framework. This design ensures consistent data representation across tasks, improves interpretability, and enables efficient analysis of very large datasets, including tens of millions of cells.

A recent trend is the integration of external biological knowledge to improve the interpretability of latent spaces. GLUE,^[^ ^69^ ^]^ for instance, employs GNNs to model regulatory interactions, linking multiple omics layers into a unified embedding. Other approaches incorporate pathway knowledge, either by constraining network architectures^[^ ^70^ ^]^ or by validating inferred interaction networks with perturbation data.^[^ ^71^ ^]^ Taking a different approach, sciLaMA^[^ ^72^ ^]^ integrates gene embeddings from LLMs with scRNA‐seq data through a paired‐VAE, enabling joint contextual representations of cells and genes for dimension reduction.

The evolution of these models reflects a shift from simple DR to integrating external knowledge—including domain‐specific priors such as regulatory networks and large‐scale representations learned by LLMs—to generate more biologically meaningful and interpretable low‐dimensional embeddings.

#### 3.1.3 Doublet Removal

A common technical artifact in droplet‐based scRNA‐seq is doublets, where two or more cells are co‐encapsulated and sequenced as one.^[^ ^13^ ^]^ Such chimeric profiles introduce spurious cell type or states, confounding downstream analyses. While established tools such as Scrublet and DoubletFinder are widely used for doublet detection.^[^ ^73^, ^74^ ^]^ AI‐driven methods—particularly deep generative models—have become instrumental by learning manifold of genuine single‐cell profiles and flagging anomalous deviations.^[^ ^13^, ^75^ ^]^

The prevailing strategy is semi‐supervised deep learning, in which models are trained to distinguish real cells from In Silico‐simulated doublets.^[^ ^76^ ^]^ In this setting, a set of labeled examples (real and simulated cells) guides the training process, while the model also leverages a much larger scale of unlabeled data to refine its decision boundary, thereby improving robustness and generalizability. Solo^[^ ^76^ ^]^ exemplifies this strategy by learning a shared low‐dimensional embedding of real and simulated doublet profiles within a VAE, followed by a classifier that distinguishes experimental doublets. This achieves 10–17% Average Precision (AP) improvement over DoubletFinder and Scrublet by leveraging the VAE's capacity to model nonlinear gene expression relationships, overcoming their limitations, and significantly improving accuracy.

### 3.2 Batch Effect Correction

Batch effects—non‐biological technical variations arising from differences in protocols, platforms, or sequencing runs—pose a central challenge in scRNA‐seq data integration.^[^ ^17^ ^]^ These artifacts can distort results by clustering cells according to batch origin rather than biological similarity, thereby obscuring meaningful signals. Consequently, effective batch correction is essential, with AI‐based methods aiming to remove batch‐specific variability while preserving biological heterogeneity. Among these, generative models are well established and widely used,^[^ ^21^, ^66^, ^77^, ^78^ ^]^ meanwhile, other innovative approaches continue to emerge.^[^ ^62^, ^79^, ^80^, ^81^, ^82^, ^83^, ^84^, ^85^, ^86^, ^87^, ^88^, ^89^, ^90^, ^91^ ^]^

The VAE paradigm underpins many widely adopted methods for data integration. A prominent example is the scvi‐tools library, which provides a suite of deep probabilistic models.^[^ ^92^ ^]^ Its core model, scVI, incorporates batch identity as a covariate in the generative process, yielding a latent space where technical variation is reduced.^[^ ^21^ ^]^ Extensions include scANVI, which uses partial cell type labels for semi‐supervised alignment,^[^ ^78^ ^]^ and totalVI, which jointly models RNA and protein measurements while correcting batch effects.^[^ ^66^ ^]^

Although these VAE‐based methods incorporate batch information as a covariate; they only implicitly mitigate batch effects without explicit disentanglement. To address this limitation, adversarial learning is introduced to explicitly separate batch effects from biological signals. Methods such as AIF and Portal adopt this strategy, enabling more effective large‐scale atlas integration.^[^ ^79^, ^80^, ^84^, ^86^, ^89^ ^]^

Another emerging direction integrates batch effect correction with downstream analytical tasks, most notably clustering.^[^ ^62^, ^81^, ^82^, ^85^ ^]^ Rather than treating correction as a separate step, these models use the clustering objective to guide correction, thereby preserving subtle biological variations and yielding more accurate and meaningful cell clusters.

Complementing these approaches, other innovative methods have demonstrated outstanding batch effect correction capabilities.^[^ ^83^, ^87^, ^88^, ^90^, ^91^ ^]^ A notable example is scArches, which employs transfer learning framework to efficiently map new datasets onto a pre‐trained reference without costly retraining, enabling decentralized atlas construction.^[^ ^83^ ^]^

The diversity of AI‐based tools reflects the complexity of batch effect correction. Among these, two strategies have proven particularly effective: explicitly conditioning latent space on batch identity, as implemented in the VAE framework of scvi‐tools, and enforcing batch invariance through adversarial learning. Specialized methods such as scArches for atlas construction and BERMAD for preserving heterogeneity further illustrate the field's progression toward more robust, experiment‐aware solutions.

### 3.3 Cell Type Identification

Cell type identification and annotation represent core objectives of scRNA‐seq. AI profoundly transforms two fundamental steps in this process: unsupervised cell clustering and automated cell annotation.

#### 3.3.1 Cell Clustering

Unsupervised clustering is a fundamental exploratory step for discovering distinct cell populations. While classical algorithms (e.g., leiden, louvain) can be applied to scRNA‐seq data,^[^ ^93^ ^]^ deep learning methods excel at handling noise and sparsity by learning specialized low‐dimensional representations that enhance cluster separability.^[^ ^21^, ^56^, ^66^, ^67^, ^78^, ^83^ ^]^ Many preprocessing models discussed earlier serve this clustering purpose, including scvi‐tools, SAUCIE, scDHA, and scArches.^[^ ^21^, ^56^, ^66^, ^67^, ^78^, ^83^ ^]^

Another strategy employs integrated deep clustering models that simultaneously optimize representation learning and cluster assignment in an end‐to‐end framework. These models embed clustering‐specific loss functions—typically based on Kullback‐Leibler (KL) divergence—into AEs, coupling feature learning with clustering.^[^ ^62^, ^81^, ^82^, ^94^ ^]^ scDeepCluster exemplifies this approach, combining a ZINB‐based AE with deep embedded clustering to achieve near‐perfect cell type identification (NMI≈1) under 30% dropout rates and robust performance (> 0.85 NMI) across 100 000 cells.^[^ ^95^ ^]^

In summary, an effective deep learning strategy for cell clustering involves joint optimization of representation learning and cluster assignment within a unified model. This approach typically combines a noise‐aware AE with a clustering‐specific loss, producing latent spaces explicitly structured for cluster separability rather than mere data reconstruction. As demonstrated by scDeepCluster, this integrated design consistently yields accurate and biologically meaningful cell populations.

#### 3.3.2 Cell Type Annotation

Following cell clustering, the next key step is cell type annotation. Traditionally, this relied on manual curation using marker genes—a process that is time‐consuming, subjective, and difficult to scale. AI has transformed this process by automating label transfer from well‐characterized reference datasets, making annotation faster, more consistent, and applicable to large datasets.^[^ ^96^ ^]^ These AI strategies span traditional supervised and semi‐supervised architectures^[^ ^97^, ^98^, ^99^, ^100^, ^101^, ^102^, ^103^, ^104^, ^105^, ^106^, ^107^, ^108^, ^109^, ^110^ ^]^ to the emerging use of large language models.^[^ ^111^, ^112^, ^113^, ^114^ ^]^

The robustness of large‐scale supervised classifiers stems from their pre‐training on vast cell atlases, enabling them to learn generalizable biological features that transcend tissue‐ and batch‐specific variations. One such method is scTab, which uses a feature‐attention model trained on over 22 million cells for robust cross‐tissue annotation, achieving Macro F1‐scores of 0.44–0.82 across 15 organs.^[^ ^100^ ^]^ However, a key limitation of supervised methods is their inability to identify cell types absent from reference data. To address this challenge: Cell BLAST^[^ ^97^ ^]^ learns a batch‐corrected embedding with a generative model and maps query cells using a tailored similarity metric while detecting uncharacterized cell types.

Deep metric learning offers further refinement by explicitly shaping the discriminative latent space.^[^ ^101^, ^104^ ^]^ TripletCell exemplifies this with triplet loss that pulls same‐type embeddings together while pushing different‐type apart, achieving clearer separation and robustness demonstrated by top cross‐sample, cross‐protocol (Macro F1 > 0.90), and cross‐species (highest scores) performance.^[^ ^104^ ^]^

Another emerging trend adopts NLP‐inspired architectures to handle the long‐tail distributions common in single‐cell data.^[^ ^99^, ^107^, ^108^, ^109^ ^]^ Celler, designed as a genomic language model, substantially improves the identification of rare cell populations often overlooked by standard models.^[^ ^109^ ^]^

Recently, LLMs built on Transformer architectures have emerged as dominant paradigms for cell annotation, achieving broad generalizability through large‐scale training. Custom‐built Transformers such as TOSICA enable interpretable annotation by explicitly incorporating biological entities such as pathways and regulons.^[^ ^111^ ^]^ Building on these advances, FMs leverage broader prior knowledge; for example, RegFormer integrates GRN hierarchies into a Mamba‐based backbone to improve both interpretability and predictive performance.^[^ ^114^ ^]^ Most notably, general‐purpose LLMs have been adapted for annotation—GPTCelltype demonstrates that models such as GPT‐4 can directly infer cell identities from annotated gene lists, offering a lightweight yet powerful alternative to conventional pipelines.^[^ ^113^ ^]^

### 3.4 AI for Trajectory Inference and Pseudotime Analysis

Understanding cells transition through continuous biological processes is essential for studying development and disease. However, scRNA‐seq data capture only static snapshots of cellular states, making trajectory inference (TI) a critical yet challenging task.

A pivotal concept for trajectory inference is RNA velocity, which infers cell's future state from its spliced and unspliced mRNA counts. Deep learning significantly enhances RNA velocity analysis. DeepVelo, for instance, extends RNA velocity to complex, multi‐lineage systems where traditional methods (e.g., Velocyto, scVelo) often fail.^[^ ^112^ ^]^ For more reliable analysis, veloVI provides VAE‐based framework that not only estimates velocities but also quantifies transcriptome‐wide uncertainty for inferred dynamics.^[^ ^115^ ^]^

Beyond models relying on splicing kinetics, deep learning architectures infer trajectories directly from gene expression patterns. scTour learns the vector field of cellular transitions and provides interpretability mechanisms to reveal driver genes via VAE.^[^ ^116^ ^]^ Similarly, VITAE combines a VAE with a latent hierarchical mixture model, enabling joint trajectory inference from multiple datasets and robust uncertainty quantification.^[^ ^117^ ^]^ While some approaches employ optimal transport (OT) to model cellular dynamics,^[^ ^118^, ^119^, ^120^ ^]^ these are less central to deep learning‐based frameworks.

Collectively, these developments have advanced TI from simple pseudo temporal ordering toward more mechanistic modeling based on diverse principles (**Table** **1**). DeepVelo extends RNA velocity to complex multi‐lineage systems, while subsequent approaches employ diverse deep learning architectures—including VAEs (scTour^[^ ^116^ ^]^) and optimal transport‐based methods (TrajectoryNet^[^ ^120^ ^]^)—to infer celluar dynamics directly.^[^ ^112^ ^]^ More recently, gene‐centric methods such as GeneTrajectory^[^ ^118^ ^]^ trace trajectories of gene programs rather than cells, disentangling overlapping process. Together, these models illustrate a progression toward predictive models of cellular dynamics.

**Table 1:** Comparison of major AI‐driven trajectory inference paradigms.

Paradigm	Core Assumptions	Excels at	Fails at
RNA Velocity‐based (e.g., DeepVelo, veloVI)	Dynamics are captured by the transient state of unspliced vs. spliced mRNA. Vectors represent the future state of a cell.	1. Inferring Local Directionality: Deduces local direction of cell fate without time‐series data. 2. Data‐Driven Rooting: Can identify trajectory origins based on the vector field. 3. Fine‐grained Dynamics: Deep learning models can capture complex transcriptional kinetics.	1. Data Quality Dependence: Highly dependent on high‐quality reads of unspliced/spliced mRNA. 2. Pseudotime Identifiability: Cannot estimate “speed” or true developmental time, only direction. 3. Smoothing Reliance: Relies heavily on k‐NN graph smoothing, which risks circular logic and artifacts.
Optimal Transport (OT)‐based (e.g., TrajectoryNet, TIGON)	Cell development follows a path of minimal cost (i.e., optimal transport). Models cell distributions at different times as probability masses to be transported.	1. Handling Time‐Series: Explicitly designed to couple samples from discrete time points. 2. Probabilistic Mapping: Quantifies probabilities and uncertainty in ancestor‐descendant relationships. 3. Global Optimality: Finds a globally optimal transport plan, not just local dynamics.	1. Computational Cost: Poor scalability; computation becomes prohibitive with large cell numbers. 2. Cost Function Sensitivity: Results are highly sensitive to the chosen “cost” definition (e.g., Euclidean distance). 3. Requires Discrete Time: Difficult to apply to steady‐state data without clear time labels.
Vector‐field VAE (e.g., scTour, VITAE)	Cell development is governed by a continuous vector field learned in a low‐dimensional latent space (by a VAE).	1. Global, Continuous Modeling: Learns a global, continuous trajectory in a unified latent space. 2. Complex Topologies: Adept at representing complex branches, cycles, and disconnected lineages. 3. Generative Capability: Can be used for in silico perturbation or cell fate prediction.	1. “Black Box” Nature: The VAE and vector field learning process can lack biological interpretability. 2. Latent Space Distortion: The learned latent space may not perfectly capture the true developmental manifold. 3. Risk of Over‐smoothing: Prone to generating overly smooth vector fields, obscuring fine local structures.

However, the application of these advanced TI models must be contextualized by the significant evaluation pitfalls inherent to the field. For traditional TI methods, topology recovery and rooting remain formidable challenges. No single algorithm has proven universally superior across all trajectory topologies (e.g., linear, bifurcating, or multifurcating); methods frequently overestimate or underestimate the complexity of the underlying biological process.^[^ ^121^ ^]^ Furthermore, many non‐velocity methods lack intrinsic directionality and require a priori biological knowledge to define a root or start cell. An incorrect choice of root can reverse the inferred progression, leading to fundamentally flawed conclusions.^[^ ^121^ ^]^

While RNA velocity‐based models (such as DeepVelo^[^ ^112^ ^]^ and veloVI^[^ ^115^ ^]^) address the rooting problem by providing an intrinsic vector field, they introduce new and more subtle interpretative challenges, particularly concerning pseudotime identifiability.^[^ ^122^ ^]^ A critical limitation is the inability to accurately estimate the “speed” of cellular transitions. The vector lengths visualized in low‐dimensional embeddings (e.g., UMAP) show little to no correlation with the actual high‐dimensional rate of gene expression change.^[^ ^122^ ^]^

More fundamentally, standard RNA velocity analysis is susceptible to circular logic, as its calculations and visualizations rely heavily on k‐nearest‐neighbor (k‐NN) graph smoothing. The resulting vector field may, therefore, represent an interpolation of the existing k‐NN structure—which also underlies the UMAP visualization itself—rather than a true extrapolation of a predictive future state.^[^ ^122^ ^]^ This dependency makes the vector field highly sensitive to the local neighborhood graph. For instance, artifactual vector field directions can be generated simply by altering the available cell populations in the low‐dimensional embedding (e.g., removing a target cell type), even when the high‐dimensional velocity estimates remain unchanged. This highlights the risk of misleading interpretations, especially if the k‐NN graph is distorted by high noise levels or sparse sampling.^[^ ^122^ ^]^

### 3.5 AI for Gene Regulatory Network Inference

Gene Regulatory Network (GRN) provides crucial insights into cellular function. AI increasingly tackles this task by learning complex dependencies directly from expression data. Several key approaches drive major advancements: multi‐omics data integration, specialized and interpretable architectures, pretrained foundation and language models that incorporating prior biological knowledges, and cutting‐edge generative models.^[^ ^123^ ^]^

Multi‐omics integration has become a powerful trend, particularly the combining of scRNA‐seq with scATAC‐seq that provides chromatin accessibility information. CellOracle exemplifies this approach, inferring GRNs and performs in silico perturbation simulations to predict the functional consequences of TF activity.^[^ ^124^ ^]^ Crucially, these in silico predictions have been validated against gold‐standard experimental perturbation data. For instance, in mouse haematopoiesis, CellOracle's in silico knockout of Cebpa accurately predicted the differentiation block at the GMP stage and the promotion of early erythroid differentiation, a result that recapitulated the cell distribution observed in ground‐truth scRNA‐seq data from actual Cebpa KO mice.^[^ ^124^ ^]^ LINGER enhances this approach by incorporating atlas‐scale bulk genomics data and TF motif knowledge as a regularization.^[^ ^125^ ^]^ Other deep learning models, such as scMultiomeGRN uses modality‐specific aggregators and cross‐modal attention,^[^ ^126^ ^]^ while scMTNI employs multi‐task learning to infer cell‐type‐specific GRNs along developmental lineages.^[^ ^127^ ^]^

Specialized and interpretable architectures represent another important direction. DeepSEM pioneered the use of a deep generative model to generalize linear structural equation models (SEMs) for GRN inference.^[^ ^123^ ^]^ GRN‐VAE, in turn, improves upon DeepSEM's stability and efficiency,^[^ ^128^ ^]^ while GRANGER employs a recurrent VAE to infer causal relationships from time‐series scRNA‐seq data.^[^ ^129^ ^]^ Addressing the critical “black box” problem, scGeneRAI applies explainable AI (XAI) techniques such as Layer‐wise Relevance Propagation (LRP) to derive interpretable, cell‐specific regulatory networks.^[^ ^130^ ^]^

Large‐scale language and FMs have recently been introduced into GRN inference to provide richer priors and capture broader biological context.^[^ ^131^, ^132^, ^133^ ^]^ By leveraging embeddings trained on massive genomic or textual data, these models supply informative representations that improve the accuracy and generalizability of inferred regulatory networks across diverse settings. Cutting‐edge generative models are driving the newest wave of innovation. Diffusion models conceptualize network inference as a reversible denoising process: DigNet uses a discrete diffusion model with a focus on global network architecture,^[^ ^134^ ^]^ while RegDiffusion employs a simpler design that learns to predict the diffusion noise.^[^ ^135^ ^]^ Advanced Transformer‐based approaches like GRNFormer further extend this line of work with a graph transformer pipeline to infer regulatory relationships.^[^ ^136^ ^]^

The evolution of AI tools reveals a multi‐pronged strategy for GRN inference. One major directions focuses on multi‐omics integration for physically‐grounded inference (e.g., CellOracle, LINGER). A second involves specialized architectures that capture dynamics and causal relationships while improving interpretability (e.g., GRANGER, scGeneRAI). A third leverages FMs to incorporate external knowledge (e.g., scRegNet), and a fourth utilizes the generative frameworks such as diffusion models and transformers (e.g., DigNet, GRNFormer).

### 3.6 AI for Cross‐Species Analysis

Cross‐species analysis is a specialized form of dataset integration, sharing methodological principles with batch correction but introducing additional biological complexity. It is central to evolutionary biology and key for translating insights from model organisms to human therapeutics. However, such analyses face challenges, including accurately mapping orthologous genes and accounting for evolutionary divergence in gene function and regulation. AI proves instrumental in overcoming these hurdles, starting from the adaptation of general integration models and evolving toward specialized frameworks that leverage fundamental biological principles.^[^ ^17^ ^]^

Early approaches adapt general integration frameworks by treating species as a “batch” variable. For instance, scVI models species identity as a covariate, establishing a shared latent space.^[^ ^21^ ^]^ Similarly, scGen—though originally developed for perturbation prediction—demonstrates that latent space vector arithmetic can simulate cross‐species responses, suggesting its features capture conserved biological variation.^[^ ^77^ ^]^ scArches further employs transfer learning to map new species data onto existing references without full retraining, offering a more scalable strategy.^[^ ^83^ ^]^

Most existing methods rely solely on gene orthology, while newer AI models incorporate deeper biological principles for robust cross‐species integration.^[^ ^137^, ^138^, ^139^, ^140^ ^]^ Protein Language Models (PLMs) represent one such key advance, capturing structural and functional protein properties from large‐scale sequence data. SATURN, for instance, integrates PLM‐derived embeddings with RNA expression profiles to align cells across species based on functional protein similarity, which is often more conserved than gene sequences.^[^ ^140^ ^]^ Harnessing a different methodology, CAME applies heterogeneous GNNs to directly assign cell types across species from single‐cell RNA‐seq data, providing quantitative assignment probabilities even for non‐model organisms.^[^ ^139^ ^]^ Another powerful strategy is to learn directly from the genomic sequence. Nvwa predicts cell‐specific gene expression from DNA sequences, thereby identifying conserved regulatory programs underlying homologous cell types across species.^[^ ^138^ ^]^

These methods reveal a clear evolutionary trajectory: from adapting general‐purpose models (e.g., scVI, scGen), to specialized reference‐mapping frameworks (scArches), and finally to approaches embedding fundamental biological priors—protein function (SATURN), gene homology networks (CAME), and DNA regulatory code (Nvwa). Building on this momentum, the field now transitions toward large‐scale, cross‐species FMs such as GeneCompass,^[^ ^34^ ^]^ marking a new era in cross‐species analysis (**Table** **2**).

**Table 2:** Summary of task‐specific AI tools in scRNA‐seq analysis.

Tool	Application	Model	Supervision	Features	Key Metrics
scGNN^[^ ^22^ ^]^	Denoising and imputation	Graph Neural Network with Multi‐modal Autoencoders	Unsupervised	Explicitly models cell‐cell relationships in a graph to inform imputation by aggregating information from neighboring cells.	Input: omics Data scale: >10k cells Evaluation metrics: ARI: 0.67–0.92 Pearson's: 0.95
scVGAE^[^ ^45^ ^]^	Denoising and imputation	Variational Graph Autoencoder (VGAE) with ZINB Loss	Unsupervised	Integrates Graph Convolutional Networks into a ZINB‐based VAE framework to preserve cell‐cell similarity during imputation.	Input: omics Data scale: 1014–22 770 cells Evaluation metrics: ARI: 0.184–0.797
DeepImpute^[^ ^53^ ^]^	Denoising and imputation	Divided Deep Neural Networks	Unsupervised	Fast and scalable "divide‐and‐conquer" strategy that learns gene‐gene relationships to predict missing values.	Input: omics Data scale: 100–50 k cells Evaluation metrics: Pearson: 0.880–0.884
DCA^[^ ^54^ ^]^	Denoising and imputation	Autoencoder with ZINB Loss	Unsupervised	Specifically models scRNA‐seq count distribution, overdispersion, and dropout rates simultaneously; highly scalable.	Input: omics Data scale: 2000 cells Evaluation metrics: Pearson's: 0.8 Spearman: 0.51
AutoClass^[^ ^55^ ^]^	Denoising and imputation	Autoencoder with an integrated Classifier	Self‐supervised	Distribution‐agnostic model that can effectively clean a wide range of noise types beyond dropouts without strong statistical assumptions.	Input: omics Data scale: 182–7162 cells Evaluation metrics: MSE: 0.5–0.6 ARI: 0.37–0.86 NMI: 0.39–0.82
scDHA^[^ ^56^ ^]^	Denoising and imputation/Cell clustering	Hierarchical Autoencoder	Unsupervised	Provides a fast, precise, and complete analysis pipeline for robust feature extraction, denoising, and downstream analysis.	Input: omics Data scale: 90–61 000 cells Evaluation metrics: R^2^: 0.93 ARI: 0.81 NMI:0.39–0.82
SERM^[^ ^57^ ^]^	Denoising and imputation	Neural Network with Data Self‐Consistency	Unsupervised	Recovers high‐fidelity expression values by learning from partial data and enforcing self‐consistency, offering high computational efficiency.	Input: omics Data scale: 2000–599 926 cells Evaluation metrics: Pearson > 0.9 Accuracy> 0.8 NMI> 0.75
scNET^[^ ^58^ ^]^	Denoising and imputation	Dual‐view Graph Neural Network	Unsupervised	Integrates external biological knowledge (Protein‐Protein Interaction networks) to learn context‐specific gene and cell embeddings for improved imputation.	Input: omics Data scale: 799–65 960 cells Evaluation metrics: AUPR: 0.65–0.97 ARI: 0.8–0.97
cnnImpute^[^ ^59^ ^]^	Denoising and imputation	1D Convolutional Neural Network (CNN)	Unsupervised	Uses a CNN to first predict dropout probability and then restore expression values, effectively capturing local gene patterns.	Input: omics Data scale: 320–4700 cells Evaluation metrics: AUPR:0.65–0.97 ARI: 0.8–0.97
scAMF^[^ ^60^ ^]^	Denoising and imputation	Manifold Fitting Module	Unsupervised	Denoises data by unfolding its distribution in the ambient space, causing cells of the same type to aggregate more tightly.	Input: omics Data scale: 10^3^–10^5^ cells Evaluation metrics: ARI: 0.78 Accuracy: 57%→ 100%
DGAN^[^ ^61^ ^]^	Denoising and imputation	Deep Generative Autoencoder Network	Unsupervised	A variational autoencoder variant that robustly imputes data dropouts while simultaneously identifying and excluding outlier cells.	Input: omics Data scale: 1000–5000 cells Evaluation metrics: ARI: 0.92 FMI: 0.89 Accuracy: 0.96
ZILLNB^[^ ^62^ ^]^	Denoising and imputation/Batch effect correction/Cell clustering	ZINB Regression with a Deep Generative Model	Unsupervised	Combines a ZINB likelihood with a deep generative model to explicitly handle zero inflation and overdispersion, producing denoised/imputed expression and a biologically meaningful latent space that supports high‐quality cell clustering, while incorporating batch covariates to correct technical variation.	Input: omics Data scale: 10^4^ cells Evaluation metrics: ARI: 0.85–0.90 Accuracy: ≈0.9
UniVI^[^ ^63^ ^]^	Denoising and imputation	Mixture‐of‐experts β‐VAE	Unsupervised	Denoises and imputes data across different modalities (e.g., scRNA‐seq, scATAC‐seq) via manifold alignment.	Input: omics Data scale: 10^4^–10^5^cells Evaluation metrics: ARI> 0.9 R^2^: 0.85–0.9
SCDD^[^ ^64^ ^]^	Denoising and imputation	Cell‐similarity diffusion + GCN‐Autoencoder denoising	Unsupervised	A two‐stage approach that first uses cell similarity for initial imputation and then a GCN‐autoencoder to denoise the result and mitigate over‐smoothing.	Input: omics Data scale: 10^2^–10^6^cells Evaluation metrics: ARI: 0.5–0.975 R^2^: 0.999 MSE: 0.061
scIDPMs^[^ ^65^ ^]^	Denoising and imputation	Conditional Diffusion Probabilistic Model	Unsupervised	Performs targeted imputation by first identifying likely dropout sites and then inferring values, which helps avoid altering true biological zeros.	Input: omics Data scale: 10^4^cells Evaluation metrics: ARI: 0.98 NMI:0.98 F–score:0.99
scVI^[^ ^21^ ^]^	Dimension reduction/Batch effect correction/Cross‐Species Analysis/Cell clustering	VAE with ZINB loss function	Unsupervised	Generates a harmonized probabilistic latent representation that disentangles biological signals from batch effects (including species) via covariate modeling, yielding denoised embeddings for high‐quality clustering.	Input: omics Data scale: 3000–1.3 M cells Evaluation metrics: ASW: 0.47 ARI: 0.81 NMI: 0.72 BE: 0.6
scGAE^[^ ^44^ ^]^	Dimension reduction	Graph Autoencoder (GAE)	Unsupervised	Explicitly preserves the topological structure of the cell‐cell similarity graph, improving trajectory inference and cluster separation.	Input: omics Data scale: 10 000 cells Evaluation metrics: NMI: 0.61–0.65
totalVI^[^ ^66^ ^]^	Dimension reduction/Batch effect correction/Cell clustering	VAE for Multi‐modal Data	Unsupervised	Jointly models RNA and surface proteins to create a unified latent space for multi‐omic analysis, simultaneously corrects batch effects in both modalities, and enables high‐quality indirect cell clustering by providing robust, denoised, integrated embeddings.	Input: omics and CITE–seq Data scale: 32 648 cells Evaluation metrics: MAE: 0.8 AUC: 0.99 Latent Mixing Metric: –0.025
SAUCIE^[^ ^67^ ^]^	Dimension reduction/Cell clustering	Deep Sparse Autoencoder	Unsupervised	Performs multiple tasks simultaneously (dimensionality reduction, clustering, imputation, batch correction) within a single, unified framework.	Input: omics Data scale: 11 million cells Evaluation metrics: Modularity: 0.8531 AUC: 0.9342
SIMBA^[^ ^68^ ^]^	Dimension reduction	Multi‐entity Graph Embedding	Unsupervised	Co‐embeds cells and their defining features (e.g., genes) into a shared latent space, enabling a unified framework for diverse tasks like marker discovery and integration.	Input: omics Data scale: million cells Evaluation metrics: ARI: 0.6 –0.9
GLUE^[^ ^69^ ^]^	Dimension reduction	Graph‐linked VAEs with Adversarial Alignment	Supervised	Accurately integrates unpaired multi‐omics data by explicitly modeling regulatory interactions with a guidance graph, ensuring scalability and robustness.	Input: omics Data scale: > 17 000 cells Evaluation metrics: ARI: 0.716 FI Score: 0.802 AMI: 0.778
Pathway‐Constrained DNNs^[^ ^70^ ^]^	Dimension reduction	Deep Neural Network with Biologically‐informed Architecture	Unsupervised	Enhances biological interpretability and reduces model complexity by designing network layers to correspond to known biological pathways.	Input: omics Data scale: Millions of cells Evaluation metrics: ASW: 0.6–0.7 R^2^: 0.236
CellBox^[^ ^71^ ^]^	Dimension reduction	ODE‐based Dynamic Systems Model	Supervised	Predicts cellular responses to unseen perturbations by learning a *de novo*, interpretable network of molecular interactions directly from data, without relying on prior pathway knowledge.	Input: omics Data scale: 100 proteins Evaluation metrics: Pearson's Correlation: 0.93
sciLaMA^[^ ^72^ ^]^	Dimension reduction	Paired‐VAE with LLM Gene Embeddings	Unsupervised	Integrates static gene embeddings from LLMs to generate context‐aware representations for both cells and genes, improving performance while maintaining computational efficiency.	Input: omics Data scale: 14 k cells Evaluation metrics: NMI: 0.745 ASW: 0.535 BatchASW: 0.865
Vaeda^[^ ^75^ ^]^	Doublet removal	Cluster‐aware VAE with Positive‐Unlabeled (PU) Learning	Supervised	Provides a more nuanced separation of singlets and doublets by considering cell cluster information during representation learning.	Input: omics Data scale: 12 k cells Evaluation metrics: AUPRC: 0.558 F1–score: 0.496 Precision: 0.59
Solo^[^ ^76^ ^]^	Doublet removal	Semi‐supervised VAE	Supervised	Achieves high accuracy by learning the manifold of genuine single‐cell profiles and then training a classifier to identify deviations (doublets).	Input: omics Data scale: 44 k cells Evaluation metrics: AP: 0.489 AUROC: 0.856
deepMNN^[^ ^90^ ^]^	Batch effect correction	Deep Learning with MNN and Residual Networks	Self‐supervised	Integrates the logic of Mutual Nearest Neighbors (MNN) into a deep learning framework for one‐step, multi‐batch correction.	Input: omics Data scale: 10^3^–10^5^ cells Evaluation metrics: ASW F1 Score: ≈0.565 ARI: ≈0.8
STACAS^[^ ^91^ ^]^	Batch effect correction	MNN‐based Method	Semi‐supervised	Leverages prior knowledge (cell type labels) to filter inconsistent anchors, improving the balance between batch correction and signal preservation.	Input: omics Data scale: 10^3^–10^5^ cells Evaluation metrics: Clisi > 0.6 Cell type ASW > 0.4
scGen^[^ ^77^ ^]^	Batch effect correction/Cross‐Species Analysis	VAE with Latent Space Arithmetic	Supervised	Models and removes batch effects by performing vector arithmetic on the latent representations of cells.Predicts cellular perturbation responses across species, demonstrating that latent space can bridge species differences.	Input: omics Data scale: 105 476 cells Evaluation metrics: R2: 0.85–0.95 ASW > 0.6
scANVI^[^ ^78^ ^]^	Batch effect correction/Cell clustering	Semi‐supervised VAE	Supervised	Uses partial cell‐type labels in a semi‐supervised VAE to more accurately align shared populations across batches, enabling high‐quality indirect clustering by first learning robust, denoised, and integrated latent representations.	Input: omics Data scale: 10 k cells Evaluation metrics: Weighted Accuracy: >0.8
scMEDAL^[^ ^79^ ^]^	Batch effect correction	Dual‐Autoencoder System	Unsupervised	Separately models batch‐invariant (fixed) and batch‐specific (random) effects, enhancing interpretability and enabling retrospective analysis.	Input: omics Data scale: 10^4^–10^5^cells Evaluation metrics: ASW: +0.69
ABC^[^ ^80^ ^]^	Batch effect correction	Semi‐supervised Adversarial Autoencoder	Semi‐supervised	Guided by a cell type classifier to ensure the retention of biological signals during adversarial batch correction.	Input: omics Data scale: 10^4^–10^5^cells Evaluation metrics: NMI: 0.91 Ilisi: 0.3
CarDEC^[^ ^81^ ^]^	Batch effect correction/ Cell clustering	Generative Models with Integrated Clustering	Self‐supervised	Performs clustering and batch effect removal jointly by optimizing a unified objective, producing batch‐invariant embeddings and clear cluster assignments within a generative/multi‐task framework that delineates cell subpopulations.	Input: omics Data scale: 10^3^–10^5^cells Evaluation metrics: ARI: 0.78–0.98
DESC^[^ ^82^ ^]^	Batch effect correction/Cell clustering	Deep Embedding and Clustering Models	Unsupervised	Performs batch effect correction and clustering jointly by optimizing a unified objective, co‐optimizing representation learning and cluster assignment end‐to‐end to produce batch‐invariant embeddings and more coherent cell groups.	Input: omics Data scale: 10^3^–10^6^cells Evaluation metrics: ARI: 0.919–0.970 Accuracy: 96.5% KL divergence: 0.6
scArches^[^ ^83^ ^]^	Batch effect correction/Cell clustering/Cross‐species analysis	Transfer Learning Framework	Supervised	Transfer‐learning maps queries to a fixed reference without retraining, providing batch‐corrected embeddings, atlas‐level clustering/label transfer, and scalable cross‐species mapping.	Input: omics Data scale: Million cells Evaluation metrics: Batch ASW: 0.5–0.7 ARI: 0.8–0.9
AIF^[^ ^84^ ^]^	Batch effect correction	Adversarial Information Factorization	Unsupervised	Factorizes batch information from the biological signal using adversarial networks, without needing prior cell type knowledge.	Input: omics Data scale: 30 K cells Evaluation metrics: ASW: 0.56–0.87 ARI: 0.89–0.91
DeepBID^[^ ^85^ ^]^	Batch effect correction	NB‐based Autoencoder with dual‐KL loss	Unsupervised	Concurrently corrects batch effects and performs clustering through an iterative process guided by a dual‐KL divergence loss.	Input: omics Data scale: 10^3^–10^6^cells Evaluation metrics: ARI: 0.65–0.97 NMI: 0.72–0.98
ResPAN^[^ ^86^ ^]^	Batch effect correction	Wasserstein GAN with Residual Networks	Unsupervised	A powerful batch correction model that combines a WGAN with mutual nearest neighbor pairing for robust integration.	Input: omics Data scale: 10^3^–10^6^cells Evaluation metrics: ARI: 0.92681 NMI: 0.90775 cLISI: 0.97093
scDML^[^ ^87^ ^]^	Batch effect correction	Deep Metric Learning	Self‐supervised	Learns a batch‐agnostic embedding space where distances between similar cells are minimized, regardless of batch origin.	Input: omics Data scale: 10^3^–10^6^cells Evaluation metrics: ARI: 0.966 NMI: 0.934
BERMAD^[^ ^88^ ^]^	Batch effect correction	Multi‐layer, Dual‐channel Autoencoder	Self‐supervised	Designed to preserve dataset‐specific heterogeneity before alignment, mitigating the risk of over‐correction.	Input: omics Data scale: 10^3^–10^5^cells Evaluation metrics: ARI: 0.94 ± 0.00
Portal^[^ ^89^ ^]^	Batch effect correction	Adversarial Domain Translation Network	Unsupervised	Fast and scalable integration that avoids over‐correction by adaptively distinguishing between shared and batch‐unique cell types.	Input: omics Data scale: 10^5^–10^6^cells Evaluation metrics: iLISI ≈1
scVAE^[^ ^94^ ^]^	Cell clustering	Generative Models with Integrated Clustering	Unsupervised	Possess integrated capabilities to delineate cell subpopulations as part of their generative or multi‐task framework.	Input: omics Data scale: 10^3^–10^6^cells Evaluation metrics: ARI: 0.656 ± 0.039
scDeepCluster^[^ ^95^ ^]^	Cell clustering	Integrated Deep Clustering (AE + KL loss)	Unsupervised	Co‐optimizes representation learning and cluster assignment in an end‐to‐end fashion for more coherent cell groups.	Input: omics Data scale: 4271 cells Evaluation metrics: ACC: 0.8100 NMI: 0.7736 ARI: 0.7841
Cell BLAST^[^ ^97^ ^]^	Cell annotation	Generative Model / Adversarial Autoencoder	Unsupervised	Provides a BLAST‐like querying system for scRNA‐seq data, using a learned, batch‐corrected embedding to annotate cells and identify novel types.	Input: omics Data scale: Million cells Evaluation metrics: MBA: 0.873
scSemiCluster^[^ ^98^ ^]^	Cell annotation	Deep Clustering with Structural Regularization	Semi‐supervised	Applies a semi‐supervised deep clustering algorithm for annotation, regularized by data structure.	Input: omics Data scale: 10^5^ cells Evaluation metrics: Accuracy:> 97% ARI: 0.95
scBalance^[^ ^99^ ^]^	Cell annotation	Sparse Neural Network with Adaptive Sampling	Supervised	Specialized tool that uses adaptive sampling techniques to enhance the identification of rare cell types.	Input: omics Data scale: 10^5^ cells Evaluation metrics: Cohen's κ: 0.95
scTab^[^ ^100^ ^]^	Cell annotation	Feature‐attention Model for Tabular Data	Supervised	A scalable model trained on over 22 million cells, achieving robust cross‐tissue annotation by focusing on relevant features.	Input: omics Data scale: 15 million cells Evaluation metrics: Macro F1: 0.7841 ± 0.0030
scVQC^[^ ^101^ ^]^	Cell annotation	Split‐vector Quantization	Supervised	The first method to apply split‐vector quantization to create discrete cellular representations that enhance cell type distinction.	Input: omics Data scale: 10^5^ cells Evaluation metrics: Accuracy: 0.86–0.95 ARI:0.82–0.88
scNym^[^ ^102^ ^]^	Cell annotation	Semi‐supervised Adversarial Neural Network	Semi‐supervised	Robustly transfers annotations across experiments by learning from both labeled reference and unlabeled query data.	Input: omics Data scale: 10^5^ cells Evaluation metrics: Accuracy: 90–92%
CAMLU^[^ ^103^ ^]^	Cell annotation	Hybrid Autoencoder + SVM	Semi‐supervised	A hybrid framework that combines an autoencoder with a support vector machine, capable of identifying novel cell types.	Input: omics Data scale: 2400–3800 cells Evaluation metrics: Accuracy: 0.95 ARI: 0.9
TripletCell^[^ ^104^ ^]^	Cell annotation	Deep Metric Learning (Triplet Loss)	Supervised	Learns a discriminative embedding space, enabling accurate annotation even across different samples or protocols.	Input: omics Data scale: 10^5^ cells Evaluation metrics: Accuracy: 80%
scDeepSort^[^ ^105^ ^]^	Cell annotation	Pre‐trained Weighted Graph Neural Network (GNN)	Supervised	An early example of a pre‐trained, weighted GNN designed for scalable and accurate cell type annotation.	Input: omics Data scale: 265 489 cells Evaluation metrics: Accuracy: 83.79% F1–score(95% CI): 0.47–0.68
mtANN^[^ ^106^ ^]^	Cell annotation	Ensemble of Models	Supervised	Improves annotation accuracy by integrating multiple reference datasets and can identify previously unseen cell types.	Input: omics Data scale: 10^5^ cells Evaluation metrics: Pearson> 0.9 AUPRC: 0.6
scGAD^[^ ^107^ ^]^	Cell annotation	Anchor‐based Self‐supervised Framework	Semi‐supervised	Solves the generalized annotation task by simultaneously annotating seen cell types from a reference and discovering/clustering novel cell types in the query data.	Input: omics Data scale: 10^5^ cells Evaluation metrics: Accuracy > 90%
CellAssign^[^ ^108^ ^]^	Cell annotation	Probabilistic Model with Marker Genes	Weakly supervised	Assigns cell types based on a predefined matrix of marker genes, making it highly effective and interpretable in specific contexts.	Input: omics Data scale: 1000–20 000 cells Evaluation metrics: Accuracy: 0.944 F1–score: 0.943
Celler^[^ ^109^ ^]^	Cell annotation	Genomic Language Model	Supervised	Specifically designed with mechanisms to address the long‐tail distribution problem for improved annotation of rare cells.	Input: omics Data scale: 10^7^ cells Evaluation metrics: F1: 0.956 Precision: 0.841± 0.002
scMMT^[^ ^110^ ^]^	Cell annotation	Multi‐use CNN Framework	Supervised	A flexible multi‐task framework that performs cell annotation alongside other tasks like protein prediction.	Input: omics Data scale: 10^5^cells Evaluation metrics: Accuracy: 0.85 ARI: 0.945
TOSICA^[^ ^111^ ^]^	Cell annotation	Transformer	Supervised	Performs interpretable annotation guided by biological entities such as pathways and regulons.	Input: omics Data scale: 647 366 cells Evaluation metrics: Accuracy: 0.8669
DeepVelo^[^ ^112^ ^]^	Trajectory Inference and Pseudotime Analysis	Deep Learning Framework	Self‐supervised	Extends RNA velocity analysis to complex, multi‐lineage systems where traditional methods often fail.	Input: omics Data scale: 10^4^ cells Evaluation metrics: Consistency Score: 0.9
GPTCelltype^[^ ^113^ ^]^	Cell annotation	Large Language Model (GPT‐4)	Self‐supervised	Demonstrates that large models can accurately infer cell types simply by interpreting lists of marker genes, automating the process.	Input: omics Data scale: 10^5^ cells Evaluation metrics: Accuracy: 0.75–0.93
RegFormer^[^ ^114^ ^]^	Cell annotation	Mamba‐based Architecture with GRN Hierarchies	Self‐supervised	A FM that integrates gene regulatory network hierarchies to enhance interpretability and performance.	Input: omics Data scale: 10^6^ cells Evaluation metrics: Accuracy: 0.86 Macro–F1: 0.77
veloVI^[^ ^115^ ^]^	Trajectory Inference and Pseudotime Analysis	Deep Generative Model (VAE)	Unsupervised	Provides crucial transcriptome‐wide uncertainty quantification for the inferred cellular dynamics, enhancing reliability.	Input: omics Data scale: 10^3^–10^4^ cells Evaluation metrics: accuracy: 66–68%
scTour^[^ ^116^ ^]^	Trajectory Inference and Pseudotime Analysis	VAE with Neural ODE	Unsupervised	Learns the vector field of cellular transitions and provides interpretability mechanisms to reveal driver genes.	Input: omics Data scale: 10^3^–10^5^ cells Evaluation metrics: Spearman ρ > 0.9
VITAE^[^ ^117^ ^]^	Trajectory Inference and Pseudotime Analysis	VAE with a Latent Hierarchical Mixture Model	Unsupervised	Enables joint trajectory inference from multiple datasets and provides robust uncertainty quantification.	Input: omics Data scale: 10^3^–10^6^ cells Evaluation metrics: ARI: 0.5–0.9 PDT: 0.4–0.9
GeneTrajectory^[^ ^118^ ^]^	Trajectory Inference and Pseudotime Analysis	Optimal Transport on a Cell‐Cell Graph	Unsupervised	A novel gene‐centric paradigm that infers trajectories of genes, allowing it to deconvolve concurrent biological programs.	Input: omics Data scale: 1000–10 500 cells Evaluation metrics: Robustness ≈ 1 Spearman: 0.9
TIGON^[^ ^119^ ^]^	Trajectory Inference and Pseudotime Analysis	Optimal Transport with Growth/Death Models	Unsupervised	Reconstructs both population dynamics and state transition trajectories simultaneously by incorporating cell growth and death.	Input: omics Data scale: 5000+ cells Evaluation metrics: Pearson: 0.62 AUROC: 0.9
TrajectoryNet^[^ ^120^ ^]^	Trajectory Inference and Pseudotime Analysis	Dynamic Optimal Transport Network	Unsupervised	Employs a dynamic optimal transport network to learn the continuous flow of cells over time.	Input: omics Data scale: 10^3^–10^5^ cells Evaluation metrics: Base TrajectoryNet: 0.897 Arch MSE: 0.300 Cycle MSE: 0.190
DeepSEM^[^ ^123^ ^]^	GRN inference	Deep Generative Model for SEMs	Unsupervised	A pioneering work that generalized linear structural equation models (SEMs) for GRN inference using a deep generative model.	Input: omics Data scale: 1000–10 500 cells Evaluation metrics: ARI: 0.82 NMI: 0.86
CellOracle^[^ ^124^ ^]^	GRN inference	GRN Inference with In Silico Perturbation	Unsupervised	Integrates scRNA/ATAC‐seq and performs in silico perturbation simulations to predict the functional consequences of TF activity.	Input: omics Data scale: 10^3^–10^5^ cells Evaluation metrics: AUROC: 0.66–0.85
LINGER^[^ ^125^ ^]^	GRN inference	GRN Inference with Regularization	Unsupervised	Enhances inference by incorporating atlas‐scale external bulk genomics data and TF motif knowledge as regularization.	Input: omics Data scale: 10^3^–10^4^ cells Evaluation metrics: AUC: 0.76 AUPR: 2.60
scMultiomeGRN^[^ ^126^ ^]^	GRN inference	Cross‐modal Attention Model	Semi‐supervised	Specifically designed for multi‐omics integration using modality‐specific aggregators and cross‐modal attention.	Input: omics Data scale: 10^3^–10^5^ cells Evaluation metrics: Accuracy: > 0.83 AUROC: 0.924 AUPR: 0.79
scMTNI^[^ ^127^ ^]^	GRN inference	Multi‐task Learning	Unsupervised	Infers cell‐type‐specific GRNs along developmental lineages from multi‐omic data.	Input: omics Data scale: 10^3^ cells Evaluation metrics: Accuracy: > 0.83 F–score > 0.3 AUPR: 0.21–0.27
GRN‐VAE^[^ ^128^ ^]^	GRN inference	VAE‐based GRN Model	Unsupervised	Improves upon the stability and efficiency of earlier generative models like DeepSEM for GRN inference.	Input: omics Data scale: 10^5^ cells Evaluation metrics: AUPRC > 1
GRANGER^[^ ^129^ ^]^	GRN inference	Recurrent VAE	Unsupervised	Infers causal relationships from time‐series scRNA‐seq data to capture the dynamic nature of GRNs.	Input: omics Data scale: 10^3^ cells Evaluation metrics: AUROC: 0.85–0.90 AUPRC: 0.90–0.98
scGeneRAI^[^ ^130^ ^]^	GRN inference	Explainable AI (XAI) Model	Unsupervised	Employs XAI techniques to infer interpretable, cell‐specific regulatory networks, addressing the "black box" problem.	Input: omics Data scale: 15 000 cells Evaluation metrics: AUC: 0.75–0.88
scGREAT^[^ ^131^ ^]^ / InfoSEM^[^ ^132^ ^]^	GRN inference	LLM‐integrated Models	Supervised	Incorporate textual gene embeddings from large language models as an informative prior to improve GRN inference.	Input: omics Data scale: thousands of cells Evaluation metrics: AUROC: 0.913 AUPRC = 0.5597
scRegNet^[^ ^133^ ^]^	GRN inference	FM+ GNN	Supervised	Combines the power of single‐cell FMs with GNNs to predict regulatory connections.	Input: omics Data scale: 800–1000 cells Evaluation metrics: AUROC: 0.93 AUPRC: 0.86
DigNet^[^ ^134^ ^]^/ RegDiffusion^[^ ^135^ ^]^	GRN inference	Diffusion Models	Unsupervised	Conceptualize network inference as a reversible denoising process, representing a new wave of generative frameworks for GRN inference.	Input: omics Data scale: thousands of cells Evaluation metrics: AUPRC: up 19–32%
GRNFormer^[^ ^136^ ^]^	GRN inference	Graph Transformer	Semi‐supervised	Uses a sophisticated graph transformer pipeline to infer regulatory relationships with high accuracy.	Input: omics Data scale: 500–5900 genes Evaluation metrics: AUROC/AUPRC: 0.90–0.98
GeneCompass^[^ ^34^ ^]^	Cross‐Species Analysis	Knowledge‐informed Transformer (FM)	Self‐supervised	A large‐scale model pre‐trained on human and mouse cells to decipher universal gene regulatory mechanisms for cross‐species tasks.	Input: omics Data scale: 126 M cells Evaluation metrics: AUC: 0.95 Annotations accuracy:0.84–0.87
CACIMAR^[^ ^137^ ^]^	Cross‐Species Analysis	Weighted Sum Model	Self‐supervised	Systematically quantifies the conservation score of cell types, markers, and interactions based on homologous features.	Input: omics Data scale: 80 777 cells Evaluation metrics: R^2^ > 0.66
Nvwa^[^ ^138^ ^]^	Cross‐Species Analysis	Deep Learning on DNA Sequences	Self‐supervised	Predicts cell‐specific gene expression from DNA sequences, allowing it to identify conserved regulatory programs across species.	Input: omics Data scale: 635 k cells Evaluation metrics: AUROC: 0.78 AUPR: 0.59
CAME^[^ ^139^ ^]^	Cross‐Species Analysis	Heterogeneous Graph Neural Network (GNN)	Self‐supervised	Directly assigns cell types across species from scRNA‐seq data and provides quantitative assignment probabilities.	Input: omics Data scale: Million cells Evaluation metrics: Accuracy: 0.87
SATURN^[^ ^140^ ^]^	Cross‐Species Analysis	Protein Language Model (PLM) Integration	Weakly supervised	Enables cell alignment based on functional protein similarity, which is often more conserved across species than gene sequences.	Input: omics Data scale: 335 000 cells Evaluation metrics: Accuracy: 0.8 ARI / NMI > 0.8

### 3.7 AI for Single‐Cell Foundation Models

The exponential growth of single‐cell RNA sequencing data offers unprecedented opportunities but also overwhelms traditional analytical methods. These conventional approaches typically build task‐specific models from scratch—a computationally intensive process that fails to leverage information shared across datasets.^[^ ^141^ ^]^ Inspired by advances in large language models, single‐cell FMs are pre‐trained on vast, diverse datasets to learn the underlying grammar of cellular function. This pre‐training yields a single, powerful model that captures generalizable representations of cell biology, serving as a versatile engine for discovery. Consequently, FMs can be efficiently fine‐tuned for diverse downstream tasks, including cell‐type annotation, perturbation prediction, and biomarker discovery. Such adaptability marks a paradigm shift from building bespoke, one‐off models to leveraging a pre‐existing knowledge base, thereby reducing computational costs and improving data efficiency.

**Figure 4:**
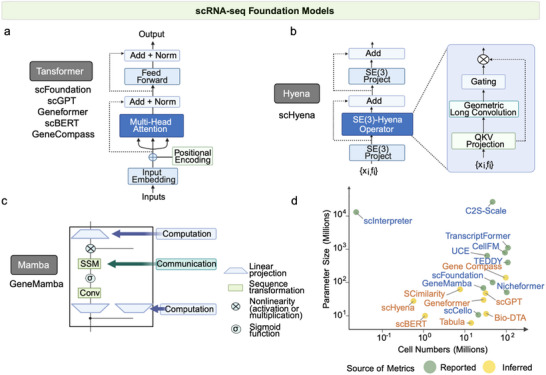
Architectures and Parameter Scaling of Foundation Models for scRNA‐seq. a) Schematic of the Transformer architecture. b) Schematic of the Hyena architecture. c) Schematic of the Mamba architecture. d) A scatter plot illustrating the scaling relationship between model parameter size (*y*‐axis) and the number of cells used for training (*x*‐axis). Green dots denote models where parameter and cell counts were explicitly reported in the publication. Yellow dots represent models where these numbers were calculated or inferred based on the descriptions in the methods section of the respective papers.

As the foundational architecture underlying most LLMs, the Transformer has become central to the development of single‐cell FMs (**Figure** **4**a). One of the earliest examples, scBERT, adapted NLP paradiagms to scRNA‐seq data for cell type annotation.^[^ ^32^ ^]^ Building on this approach, Geneformer demonstrated scalability by training on a corpus of over 30 million cells, substantially expanding model capacity.^[^ ^33^ ^]^ More recently, scGPT has emerged as a highly influential model, introducing “gene‐cell” dual embeddings and achieved strong zero‐shot transfer performance across diverse tasks such as multi‐omics integration and perturbation prediction.^[^ ^23^ ^]^

Concurrently, architectural innovation is advancing beyond standard Transformers along several complementary fronts, including computational efficiency, data‐structure awareness, and biological knowledge integration. At the efficiency frontier, GeneMamba introduces the first single‐cell FM built on the Mamba state‐space model (SSM) architecture (Figure 4c), achieving linear‐time complexity that scales favorably for long gene sequences.^[^ ^142^ ^]^ In a parallel effort, scHyena leverages the Hyena operator, another efficient attention‐free architecture (Figure 4b)—to analyze full‐length transcript data in brain tissue.^[^ ^143^ ^]^ Shifting to data‐structure considerations, Tabula pioneers an FM that explicitly models its tabular nature of single‐cell data while uniquely incorporating federated learning to preserve data privacy.^[^ ^144^ ^]^ Beyond these architectural advances, researchers are increasingly embedding deeper biological knowledge and multi‐modality information into FMs.^[^ ^140^, ^145^, ^146^, ^147^, ^148^ ^]^ For example, Nicheformer achieves a significant leap in spatial context integration by pre‐training on over 110 million dissociated and spatially resolved cells, thereby learning representations that encode the local tissue microenvironment.^[^ ^146^ ^]^ On the evolutionary front, TranscriptFormer trains a generative multi‐species model on 112 million cells across 12 species, providing a powerful framework for studying conserved biology across 1.5 billion years of evolution.^[^ ^145^ ^]^ A parallel trend involves the convergence of genomics and NLPs: scInterpreter is an LLM specifically trained to interpret scRNA‐seq data through a natural language interface,^[^ ^149^ ^]^ while the C2S‐Scale framework pushes this paradigm further by training massive LLMs (up to 27 billion parameters) on over a billion tokens of “cell sentences” and biomedical text.^[^ ^150^ ^]^ Finally, specialized FMs like SCimilarity, address targeted analytical needs, offering optimized architectures for scalable cell similarity search across massive atlases.^[^ ^151^ ^]^

Scaling has become a central theme in the field, with models expanding in both parameter count and training data size (Figure 4d). scFoundation pioneers this trend, reaching the 100 million parameter scale through pre‐training on 50 million cells and employing a unique asymmetric encoder‐decoder design to manage the large number of genes.^[^ ^152^ ^]^ Building on this momentum, the TEDDY family further advanced these limits, scaling to 400 million parameters and 116 million cells while innovatively incorporating large‐scale biological annotations as weak supervision during pre‐training.^[^ ^153^ ^]^ This benchmark is subsequently eclipsed by CellFM, which at 800 million parameters trained on 100 million human cells—establishing a new standard for handling technical noise and batch effects.^[^ ^35^ ^]^

While this race for raw parameter count continued, GeneCompass^[^ ^34^ ^]^ represents a complementary advance, emphasizing both scale and knowledge integration through its cross‐species FM trained on over 100 million human and mouse cells. It uniquely incorporates prior biological knowledge—including GRNs, promoter sequences, gene family information, and co‐expression networks—into self‐supervised pre‐training, thereby enabling the model to learn conserved regulatory mechanisms while supporting both single‐ and cross‐species applications (**Table** **3**).

**Table 3:** Summary of Foundation Models applied to scRNA‐seq data.

Tool	Model	Features	Pretrained Weight	Parameter Size	Cell Number
scGPT^[^ ^23^ ^]^	Generative Pre‐trained Transformer	A powerful generative model with a "gene‐cell" dual embedding, demonstrating strong zero‐shot transfer learning performance.	Provided	50 M	33 M
scBERT^[^ ^32^ ^]^	BERT‐style Transformer	A pioneering work that first introduced the BERT‐style deep language model for cell type annotation of scRNA‐seq data.	Provided	10 M	1.1 M
Geneformer^[^ ^33^ ^]^	Transformer	Innovatively treats genes ranked by expression as a "sentence," using self‐attention to learn gene‐gene interactions.	Provided	30 M	30 M
GeneCompass^[^ ^34^ ^]^	Knowledge‐Informed Transformer	A knowledge‐informed model that integrates four types of prior biological knowledge. Its dual‐task pre‐training decodes both the ID and absolute expression value of masked genes	Provided	142 M	101.77 M
CellFM^[^ ^35^ ^]^	Very Large Transformer (800 M parameters)	One of the largest models, setting a new standard for handling technical noise and batch effects at an unprecedented scale.	Provided	800 M	102 M
GeneMamba^[^ ^142^ ^]^	Mamba State‐Space Model (SSM)	The first single‐cell FM based on the Mamba architecture, achieving linear‐time complexity that is highly efficient for long gene sequences.	Provided	65.74 M	30 M
scHyena^[^ ^143^ ^]^	Hyena Operator (Attention‐free)	An efficient attention‐free architecture for the analysis of full‐length transcript data, particularly in complex tissues like the brain.	Not Provided	27.74 M	0.575 M
Tabula^[^ ^144^ ^]^	Tabular Data Model with Federated Learning	The first FM designed to explicitly model the tabular nature of scRNA‐seq data and uniquely incorporates federated learning to preserve data privacy.	Provided	6 M	15 M
TranscriptFormer^[^ ^145^ ^]^	Generative, Multi‐species Transformer	A powerful framework trained on 112 million cells across 12 species, designed for studying conserved biology across evolution.	Provided	1077 M	112 M
Nicheformer^[^ ^146^ ^]^	Single‐cell and Spatial omics Transformer	The first large‐scale integration of dissociated and spatial transcriptomics data, learning to predict a cell's spatial microenvironment information from its gene expression	Provided	49.3 M	110 M
scCello^[^ ^147^ ^]^	Cell Ontology‐guided Transformer	The first transcriptome FM to be guided by a cell ontology, structuring its learning process using the hierarchical relationships between cell types.	Provided	10.7 M	22 M
Bio‐DTA^[^ ^148^ ^]^	Multi‐modal FM	A pioneering multi‐modal FM that jointly learns from transcriptomes and DNA sequences via a dynamic token adaptation method.	Not Provided	11.4 M	33.4 M
C2S‐Scale^[^ ^150^ ^]^	Massive Language Model	Takes the "cell as sentence" concept to the extreme, training massive LLMs on over a billion tokens of cell sentences and biomedical text.	Provided	27 000 M	50 M
SCimilarity^[^ ^151^ ^]^	Deep Metric Learning / Neural Network	A FM specifically optimized for performing scalable and accurate cell similarity searches across massive cell atlases.	Provided	62.3 M	7.9 M
scFoundation^[^ ^152^ ^]^	Asymmetric Encoder‐Decoder Transformer	A large‐scale model with an asymmetric architecture designed to efficiently handle the large number of genes.	Provided	100 M	50 M
TEDDY^[^ ^153^ ^]^	Large‐scale Transformer (up to 400M parameters)	Innovatively uses large‐scale biological annotations as a form of weak supervision during pre‐training to enhance performance.	Not Provided	414.2 M	116 M
UCE^[^ ^154^ ^]^	Protein Language Model‐based Embedding	Utilizes a protein language model to convert genes to protein embeddings, thereby bypassing species homology limitations to achieve universal, zero‐shot cell representation.	Provided	650 M	36 M

Note: The unit for “Parameter Size” and “Cell Number” is million (M).

## 4 The Application of AI in Spatial Transcriptomics

**Figure 5:**
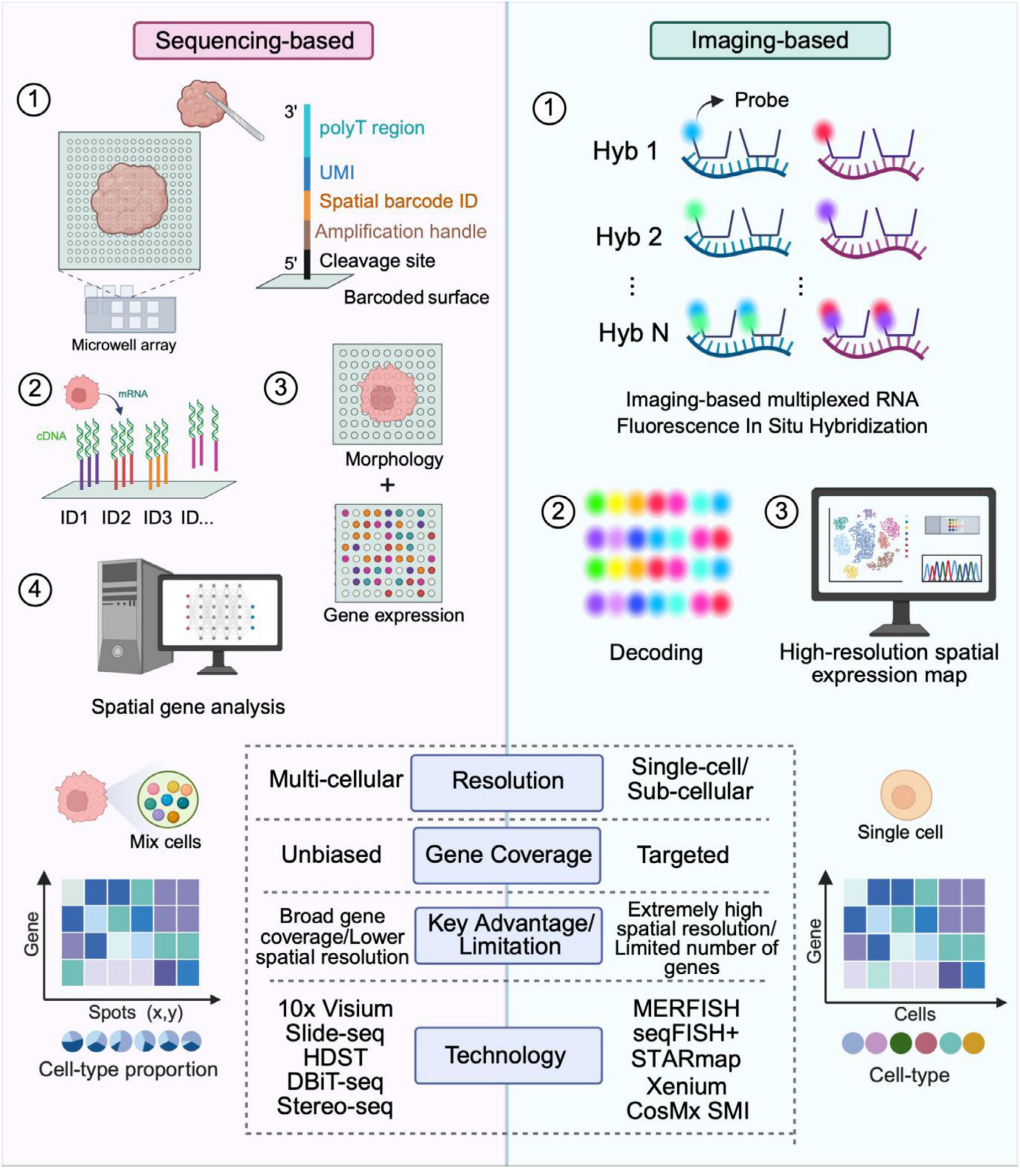
Overview of Spatial Transcriptomics Technologies: Sequencing‐based vs Imaging‐based Approaches. Sequencing‐based methods capture transcripts on barcoded surfaces for reverse transcription, amplification, and sequencing, offering unbiased and broad gene coverage but with limited spatial resolution (e.g., 10x Visium, Slide‐seq, DBiT‐seq, Stereo‐seq). Imaging‐based methods use iterative probe hybridization and fluorescence imaging to directly visualize RNA molecules in situ, enabling single‐cell or subcellular resolution with targeted gene panels (e.g., MERFISH, seqFISH+, STARmap, Xenium, CosMx).

ST technologies are characterized by an intrinsic trade‐off between gene coverage and spatial resolution, largely delineated by two main categories (**Figure** **5**). 1) Sequence‐based methods, such as 10x Visium,^[^ ^10^ ^]^ Slide‐seq,^[^ ^155^ ^]^ HDST,^[^ ^156^ ^]^ DBiT‐seq,^[^ ^157^ ^]^ and Stereo‐seq,^[^ ^158^ ^]^ offer genome‐wide transcriptomic profiling across large tissue areas. However, their lower spatial resolution means that gene expression is often captured from multi‐cellular “spots”, creating mixed signals that obscure underlying single‐cell heterogeneity. 2) Imaging‐based technologies, including MERFISH,^[^ ^159^ ^]^ STARmap,^[^ ^160^ ^]^ seqFISH+,^[^ ^161^ ^]^ Xenium, and CosMx SMI,^[^ ^162^ ^]^ achieve subcellular resolution but are restricted to smaller regions of interest and require predefined gene panels. These inherent trade‐offs, combined with technical noise across platforms and the spatial complexity of tissues, create significant data analysis challenges that AI approaches are increasingly being developed to address.^[^ ^163^ ^]^

**Figure 6:**
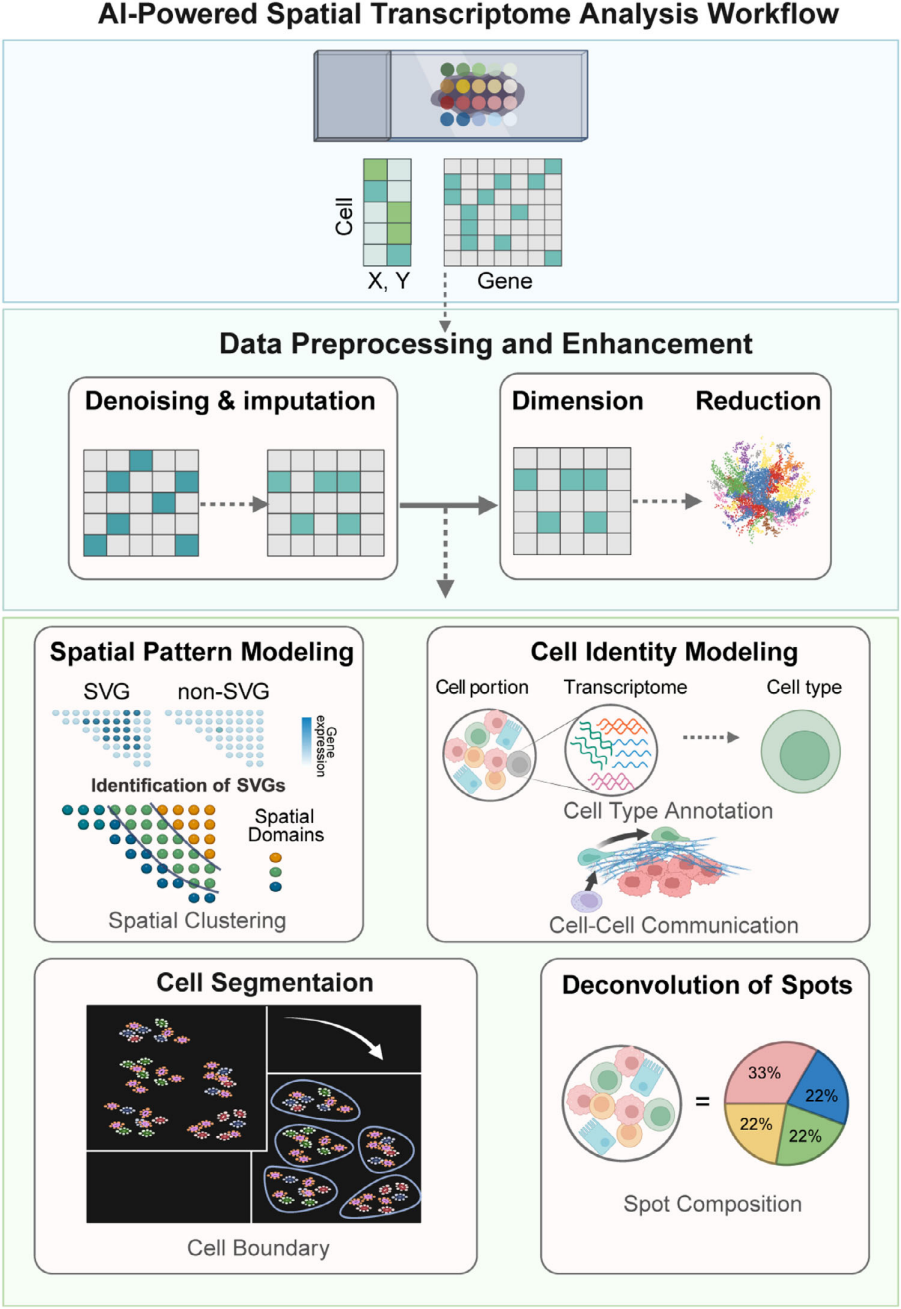
AI‐Powered Spatial Transcriptome Analysis Workflow. Data preprocessing and enhancement, including denoising, imputation, and dimension reduction to improve data quality; and downstream analytical tasks, covering spatial pattern modeling, cell identity modeling, cell segmentation, and spot deconvolution to derive biological insights from spatial transcriptomic data.

AI‐based methods are rapidly emerging to address these analytical challenges across the ST analysis pipeline, from preprocessing to downstream tasks. Here, we systematically review these applications according to a typical workflow (**Figure** **6**).

### 4.1 Data Preprocessing and Enhancement

Quality control is the first step in ST analysis, as with scRNA‐seq data, to remove low‐quality cells or spots. However, ST presents greater complexity by coupling gene expression with spatial features such as 2D or 3D coordinates and tissue images.^[^ ^164^, ^165^ ^]^ Additionally, ST data suffers from substantial noise, including dropout events, mRNA diffusion, and limited gene numbers—limitations inherent to current technologies.^[^ ^166^ ^]^ Together, these challenges render ST preprocessing particularly demanding, yet this step remains essential for reliable downstream analyses.

#### 4.1.1 Denoising and Imputation

Owing to inherent technical limitations, ST data typically exhibit high noise and sparsity, demanding computational methods that integrate spatial context—a feature lacking in standard scRNA‐seq AEs.^[^ ^167^, ^168^, ^169^ ^]^ The initial evolution of these models focus on how to represent spatial information. Graph Autoencoders (GAEs) explicitly encode spatial relationships, using a graph of spot proximities to regularize the AE's latent space.^[^ ^170^, ^171^ ^]^ For instance, SEDR^[^ ^172^ ^]^ combines an AE with a variational graph autoencoder (VGAE) to learn spatial features for improved denoising and imputation, achieving robust performance as quantified by the highest Pearson Correlation Coefficient (PCC) of 0.86 for gene expression imputation on the Mouse Olfactory Bulb dataset.

While GAEs are effective, their reliance on a discrete graph can be rigid. A more flexible alternative, STINR,^[^ ^171^ ^]^leverages an Implicit Neural Representation (INR) to instead model gene expression as a continuous function of its spatial coordinates. This approach avoids the need for a predefined graph and can theoretically handle data at arbitrary resolutions or with misaligned slices. Concurrently, other methods have focused on enhancing the generative architecture itself, such as SpaIM^[^ ^173^ ^]^ and stDiff,^[^ ^174^ ^]^ which adapt powerful style‐transfer and diffusion‐based models, respectively, to capture complex, non‐linear expression patterns.

However, all these methods primarily learn relationships *de novo* from the ST data itself. A critical challenge arises when trying to recover mechanistic insights, especially for genes with high dropout rates. If a gene's signal is almost entirely absent, spatial interpolation alone is insufficient to recover it. To overcome this, a new class of models moves from *de novo* pattern fitting to knowledge‐guided imputation by integrating external, foundational biological knowledge.^[^ ^173^, ^174^, ^175^, ^176^, ^177^, ^178^, ^179^, ^180^, ^181^ ^]^

These models tap into two primary sources of external data. The first is histology, where methods like BLEEP,^[^ ^179^ ^]^ uses a bimodal contrastive learning strategy (inspired by CLIP^[^ ^178^ ^]^) to align H&E image patches and gene expression, enabling the model to infer expression based on visible tissue morphology. The second, more fundamental, strategy is to import knowledge from LLMs pre‐trained on vast biological sequence data. This provides a prior for biologically plausible gene‐gene relationships that is independent of the experimental noise. For instance, StImpute^[^ ^181^ ^]^ constructs its graph based on protein similarity from ESM‐2^[^ ^176^ ^]^ embeddings, while spRefine^[^ ^177^ ^]^ uses embeddings from a genomic language model (gLM) within its autoencoder.

This integration of pre‐trained knowledge marks a significant shift. By grounding the imputation in functional genomics (via LLMs) or morphology (via H&E), these models can reconstruct mechanistically plausible gene networks rather than just spatially smooth patterns. The key challenge, however, now shifts from simple denoising to a more complex integration problem: how to best balance the static, universal knowledge from an LLM with the dynamic, context‐specific spatial patterns observed in a given ST experiment. Effectively uniting these two data modalities is the next frontier for extracting robust biological insights from ST data.

#### 4.1.2 Dimension Reduction

To facilitate visualization and analysis of large‐scale ST data, DR is applied to denoise signals, remove redundant information, and reduce computational burden.^[^ ^182^ ^]^ Conventional approaches such as PCA,^[^ ^183^ ^]^ NMF,^[^ ^166^ ^]^ LDA,^[^ ^184^ ^]^ and LDVAE^[^ ^185^ ^]^—originally developed for scRNA‐seq—are widely applied in ST. However, these methods operate solely on the expression matrix, and ignore the spatial coordinates. Consequently, while effective for compressing expression features, they fail to capture the inherent spatial tissue structures.

This limitation has motivated the development of spatially informed DR frameworks. Representative methods such as SpaGCN,^[^ ^42^ ^]^ GraphST,^[^ ^186^ ^]^ and BayesSpace,^[^ ^187^ ^]^ integrate spatial information through GNN or probabilistic models. Nevertheless, their latent embeddings are often opaque and require additional processing for biological interpretation. As noted in the STAMP,^[^ ^188^ ^]^ these embeddings can be “black‐box” and are typically interpreted only *post hoc* through subsequent clustering and differential expression analysis. This creates a critical gap: analysts can identify where distinct tissue domains are, but the embeddings themselves do not explicitly reveal the biological programs or gene sets driving that domain's identity.^[^ ^188^, ^189^ ^]^

Addressing the challenge of moving from spatial patterns to mechanistic biological insights requires models that produce inherently interpretable embeddings. Two recent methods, STAMP and SpaHDmap, tackle this problem using different “interpretable‐by‐design” philosophies: STAMP^[^ ^188^ ^]^ is an interpretable deep generative model for spatial transcriptomics, achieving interpretability through topic modeling of biologically meaningful gene modules while incorporating spatial context via a simplified GCN^[^ ^190^ ^]^ into the embeddings. In contrast, SpaHDmap^[^ ^189^ ^]^ derives its interpretability primarily from multimodal fusion, particularly through the integration of morphological features from histological images.

In summary, while spatially‐aware methods like GraphST and BayesSpace excel at identifying where domains are, models like STAMP and SpaHDmap are designed to also reveal what they are. STAMP achieves this through gene‐centric topic modeling, while SpaHDmap uses a parts‐based NMF approach fused with high‐resolution image morphology.

### 4.2 AI for Spatial Pattern Modeling

Recent AI advances have substantially enhanced spatial pattern modeling, particularly in two key tasks: spatial clustering and the identification of spatially variable genes (SVGs). These complementary tasks are essential for characterizing tissue organization—spatial clustering reveals distinct domains while SVG detection identifies genes with significant spatial variation.^[^ ^191^ ^]^ Together, they provide critical insights into tissue architecture and functional regionalization, laying the groundwork for diverse downstream investigations.

#### 4.2.1 Identification of SVGs

The detection of SVGs lies at the heart of deciphering functional tissue organization, as these genes reflect structured expression patterns that define anatomical regions and functional niches. Traditional statistical approaches, while robust and interpretable,^[^ ^192^ ^]^ often fail to capture or model the complex nonlinear spatial relationships inherent in the multimodal data of organizational biology.^[^ ^42^ ^]^ To overcome these limitations, AI‐based methods have emerged, offering superior sensitivity and scalability for SVG detection while leveraging multimodal data integration.^[^ ^42^, ^193^, ^194^, ^195^, ^196^ ^]^ These AI frameworks have diverged into distinct approaches, each adopting a unique fundamental assumption about how spatial gene expression is organized relative to tissue structure.

spaGCN^[^ ^42^ ^]^ and SPADE^[^ ^193^ ^]^ pioneer the multimodal fusion of H&E‐stained images with ST data. Specifically, spaGCN constructs graphs based on ST data and integrates H&E‐stained images using GCNs. The alignment of morphology between ST data and images enhances the interpretability of SVG identification. In contrast, SPADE directly leverages limma to conduct regression analysis between gene expression and image features extracted by CNNs,^[^ ^197^ ^]^ achieving morphology‐driven SVG identification.^[^ ^193^, ^198^ ^]^

Focusing on the utilization of ST data, PROST treats gene expression matrices as images to compute a foreground‐background “PI score.”^[^ ^195^ ^]^ This image analysis strategy excels in high‐resolution analyses, detecting fine‐grained structures such as the delicate branching of olfactory nerves—details often overlooked by standard clustering methods. Conversely, GASTON employs a different principle, modeling tissue as a continuous field of “isodepths.”^[^ ^194^ ^]^ This topographical framework effectively captures gradual biological processes, such as metabolic gradients, that would otherwise be artificially fragmented by cluster‐based approaches. Finally, the simple yet effective GAADE optimizes neighbor information by incorporating the first‐order and second‐order neighbors of the target node, excelling at the local identification of SVGs.^[^ ^196^ ^]^

Despite these capabilities, selecting the optimal tool for SVG identification requires balancing trade‐offs based on the research objective. In recent benchmarks,^[^ ^192^, ^199^, ^200^ ^]^ As a statistical method, SPARK‐X is highlighted as suitable for large‐scale datasets due to its linear *O(N)* complexity while outperform other models.^[^ ^201^ ^]^ Conversely, if tissue structures and spatial domains are the primary concern, spaGCN^[^ ^42^ ^]^ and SPADE^[^ ^193^ ^]^ are the preferable choices.

#### 4.2.2 Spatial Clustering

Spatial clustering represents one of the most active and methodologically diverse areas in computational ST, with numerous AI‐based models developed to date. This proliferation stems from the task's central importance in mapping tissue architecture and AI models’ ability for robust, scalable analysis of large, complex datasets.^[^ ^202^ ^]^ For clarity, we first discuss graph‐based methods,^[^ ^42^, ^203^, ^204^, ^205^, ^206^, ^207^, ^208^, ^209^ ^]^ (encompassing both GNN‐ and GCN‐derived models), followed by contrastive learning‐based^[^ ^41^, ^186^, ^210^, ^211^, ^212^ ^]^ and transformer‐based clustering strategies.^[^ ^213^, ^214^ ^]^

Graph‐based methods have established a strong foundation for spatial clustering by explicitly modeling local spatial relationships. Pioneering work such as SpaGCN^[^ ^42^ ^]^ integrates gene expression, spatial coordinates, and histological features through GCNs on undirected weighted graphs, achieving strong performance. Subsequent models have diversified architecturally: DeepST^[^ ^206^ ^]^ employs parallel dual autoencoders for denoising and spatial information, while ResST^[^ ^204^ ^]^ enhances low‐dimensional gene representations by incorporating spatial embeddings as residuals. Beyond multimodal fusion, several approaches leverage cell type information—by refining clustering, estimating cell type proportions, or strengthening latent embeddings—to improve biological interpretability and robustness across datasets.^[^ ^203^, ^207^, ^208^ ^]^ Building on these integrated strategies, MAFN^[^ ^209^ ^]^ constructs spatial and feature graphs, fuses them with a co‐graph, and applies a CAM attention module^[^ ^205^ ^]^ to balance spatial continuity with gene expression specificity, yielding clearer regional boundaries.

In contrast, contrastive learning approaches avoid explicit graph construction and instead learn representations by aligning multimodal views of the same spot or tissue region. This paradigm excels at integrating heterogeneous data types, as demonstrated by conST, which integrates ST data with histological images, ensuring consistent clustering across different levels of granularity. GraphST^[^ ^186^ ^]^ simplifies this approach by directly contrasting spatial location and gene expression embeddings. Building on these principles, STAGUE^[^ ^210^ ^]^ introduces a triple‐view contrastive learning that captures both spatial proximity and biological coherence. Other models in this category place a particular emphasis on image features: stMVC^[^ ^212^ ^]^ and STAIG^[^ ^41^ ^]^ leverage frameworks such as SimCLR^[^ ^27^ ^]^ and BYOL^[^ ^28^ ^]^ for feature extraction, with stMVC focusing on tumor heterogeneity and STAIG ensuring morphological consistency through debiased negative sampling.

A more recent trend involves the adaptation of transformer architectures, which can capture long‐range spatial dependencies and reweight interactions between spots. The Graph Transformer^[^ ^215^ ^]^ exemplifies this, dynamically updating graph topologies to capture these dependencies and offering a strong alternative to conventional GNNs and GCNs. Building on this, SiGra^[^ ^213^ ^]^ constructs graphs from multi‐channel IHC images and fuses them with ST data in a parallel framework. SpaGT^[^ ^214^ ^]^ streamlines this approach by iteratively updating both expression and edge embeddings using multi‐head self‐attention with residual connections, generating informative, less noisy embeddings for improved spatial clustering.

Overall, graph‐based, contrastive learning‐based, and transformer‐based models each contribute unique strengths to spatial pattern modeling. Graph methods emphasize local structure, contrastive approaches enhance robustness and modality integration, and transformer architectures capture long‐range dependencies. Together, they mark a shift toward more flexible and scalable strategies for uncovering tissue architecture in spatial transcriptomics.

### 4.3 AI for Cell Type Annotation

Many cell type annotation methods initially developed for scRNA‐seq analysis can be directly applied for ST.However, a fundamental limitation is that these methods are designed for dissociated cells and thus ignore spatial organization. To address this limitation, several models have been developed that incorporate spatial information, demonstrating strong performance for ST data.^[^ ^216^, ^217^, ^218^, ^219^ ^]^ These approaches typically rely on graph construction to model non‐Euclidean relationships and integrate multimodal features such as gene expression and spatial coordinates.

Due to the spot‐level resolution of ST data—which often results in mixed cellular signal within each spot—reference‐based methods are often prioritized for accurate cell type annotation. Among pioneering graph‐based approaches, STELLAR^[^ ^220^ ^]^ explicitly incorporates spatial coordinates to construct graphs and generates pseudo‐labels for unknown cell types through domain nearest neighbors, making it particularly suited for discovering novel subpopulations in previously uncharacterized tissues. Spatial‐ID,^[^ ^217^ ^]^ another graph‐based method, relies heavily on high‐quality scRNA‐seq reference data and exemplifies a classical transfer learning strategy. Most recently, Focus^[^ ^216^ ^]^ advances spatial granularity by modeling transcriptional networks at subcellular resolution with graph contrastive learning, shifting the emphasis from intercellular interactions to intracellular topology to enable more precise annotation.

Overall, integrating spatial information—particularly histology‐derived morphological features—substantially improves cell type annotation in ST compared to methods designed for dissociated scRNA‐seq data. Building on this insight, future approaches should incorporate additional modalities such as spatial proteomics and model spatial context across multiple scales to achieve more biologically interpretable results.^[^ ^221^ ^]^

### 4.4 AI for Deconvolution of Spatial Spots

Due to the limited resolution of spots in ST data, cellular deconvolution is crucial for estimating cell types proportions within each spot. Resolving cellular composition reveals the spatial organization of distinct populations and facilitates reconstruction of detailed tissue architecture.^[^ ^222^ ^]^ Existing approaches can be broadly divided into two categories: reference‐based methods^[^ ^223^, ^224^, ^225^, ^226^, ^227^, ^228^, ^229^ ^]^ that leverage external scRNA‐seq datasets, and reference‐free methods^[^ ^230^, ^231^, ^232^ ^]^ that infer composition directly from ST data.

In reference‐based AI approaches, scRNA‐seq reference data is used to generate pseudo‐ST spots (e.g., randomly sampled) for deconvolving true ST data spots—essentially simulating unknown data states using known data. Different methods employ distinct implementation principles. For instance, SD2^[^ ^224^ ^]^ considers dropout rate variations to reflect spot composition changes more directly than HVGs, making it preferable tool for addressing highly sparse datasets. STdGCN^[^ ^225^ ^]^ fuses graphs constructed from expression levels and spatial coordinates, optimized for large‐scale datasets (multiple spots, multiple cell types). In contrast, LETSmix^[^ ^229^ ^]^ introduces a comprehensive filtering mechanism integrating multiple spatial features (including layer annotations, expression similarity, and image textures). Notably, it employs a mixup‐augmented domain adaptation strategy to mitigate technical discrepancies between datasets, thereby making it suitable for cross‐platform applications.

Reference‐free methods offer an alternative strategy that estimates cell composition without relying on predefined expression profiles. These approaches typically utilize factor decomposition or probabilistic modeling to uncover latent gene programs representing distinct cell types.^[^ ^230^, ^231^, ^232^, ^233^ ^]^ As one of the earliest reference‐free methods, STdeconvolve^[^ ^230^ ^]^ employs topic modeling in a fully unsupervised manner to infer cellular components driven entirely by gene expression. However, because the resulting latent topics lack direct biological labels, they require *post hoc* annotation using marker genes and may exhibit variability across runs or struggle to capture rare cell populations. In contrast, SMART^[^ ^232^ ^]^ improves upon this topic modeling framework by adopting a semi‐supervised design that incorporates marker gene lists as prior knowledge. This marker‐gene‐assisted strategy not only eliminates the need for *post hoc* annotation but also guides the inference process to enhance the stability of predictions and the identification of less abundant cell types.

Despite the proliferation of deconvolution methods, both approaches remain fundamentally limited. Reference‐based methods must contend not only with “technology effects”—systematic biases in dropout rates and capture efficiency—but also with biological mismatches (e.g., differences in donor demographics, tissue regions, or in vitro culturing conditions) between the reference and target data.^[^ ^224^, ^229^ ^]^ Notably, benchmarking studies have shown that these biological factors often outweigh technical discrepancies in influencing deconvolution accuracy.^[^ ^233^ ^]^ Meanwhile, reference‐free methods struggle with parameter selection^[^ ^230^, ^233^ ^]^ (e.g., determining the correct number of cell types) and distinguishing transcriptionally similar subtypes.^[^ ^232^ ^]^ Furthermore, their performance relies heavily on compositional heterogeneity across samples; they often fail in datasets where cell‐type proportions are relatively homogeneous.^[^ ^230^, ^233^ ^]^

Therefore, reference‐based methods represent the optimal choice when a high‐quality scRNA‐seq dataset from biologically matched tissue is available.^[^ ^230^, ^233^ ^]^ They are most suitable for projects aiming to map known cell types with high consistency.^[^ ^225^ ^]^ Conversely, reference‐free methods are preferable when reliable reference data are unavailable or when the primary goals include discovering novel cell states that reference‐based methods might miss.^[^ ^230^, ^232^ ^]^ However, users must ensure sufficient sample‐to‐sample variation for these methods to resolve latent components effectively.^[^ ^233^ ^]^

These limitations underscore the need for advanced strategies. Domain adaptation (e.g., LETSmix) and FMs offer promising directions. The former employs deep learning mechanisms to mitigate technical and biological discrepancies between the reference and target data, thereby reducing the reliance on perfect matching. Meanwhile, the latter, trained on massive datasets, provides highly generalizable references adaptable to various biological conditions.

### 4.5 AI for Cell Segmentation

Cell segmentation quality fundamentally dictates biological interpretation, as transcript misassignment (“spillover”) can fabricate mixed phenotypes (e.g., distinguishing epithelial‐immune doublets from true interactions), obscure true cell identities, or spuriously create ligand‐receptor interactions that lead to incorrect mechanistic conclusions.^[^ ^221^, ^234^, ^235^, ^236^, ^237^ ^]^ To secure this fidelity, contemporary AI strategies have evolved into two distinct families: morphology‐based models that rely on visual inputs or transcript‐derived “pseudo‐images”,^[^ ^49^, ^50^, ^51^, ^238^ ^]^ and transcriptome‐based models that operate directly on transcript locations.^[^ ^52^, ^238^, ^239^, ^240^, ^241^, ^242^, ^243^, ^244^ ^]^

Despite these differing inputs, recent architectures offer distinct performance gains. Advanced morphology‐based models like Cellpose3 integrate restoration modules to denoise and deblur low‐quality staining input, while incorporating human‐assisted for improved performance.^[^ ^49^, ^50^, ^51^ ^]^ Transformer‐based architectures like CelloType leverage multitask learning to simultaneously segment and classify cells using global context.^[^ ^238^ ^]^ In contrast, transcriptome‐based approaches like segger^[^ ^241^ ^]^ and FICTURE^238^ utilize GNN and stochastic spatial factorization, respectively, to recover cytoplasmic transcripts and delineate irregular morphologies (e.g., adipocytes, fibroblasts) that defy standard nuclear boundaries.

However, these approaches entail distinct trade‐offs. morphology‐based methods are highly sensitive to staining quality: robust auxiliary signals enable learning of true morphology, whereas faint or unreliable staining can sharply degrade performance.^[^ ^234^ ^]^ Pseudo‐image pipelines (and standard platform outputs) mitigate staining dependence but impose a “bag‐of‐RNA” assumption that can blur subcellular localization, occasionally producing morphologically implausible boundaries or hallucinated cells.^[^ ^234^, ^235^ ^]^ Transcriptome‐based methods avoid these particular artifacts but introduce their own difficulties, primarily the challenge of inferring sharp boundaries for non‐convex or densely packed cells without morphological guidance.^[^ ^234^ ^]^

No automated method is universally reliable. Choosing between morphology‐based and transcriptome‐based segmentation entails balancing modeling assumptions, data requirements, and computational budgets. In practice, robust discovery still benefits from a human‐in‐the‐loop validation strategy that checks critical boundaries and assignments to safeguard downstream analyses (**Table** **4**).^[^ ^50^, ^234^ ^]^

**Table 4:** Summary of task‐specific AI tools in spatial transcriptomics.

Tool	Application	Model	Supervision	Features	Key metrics
SEDR^[^ ^172^ ^]^	Denoising and Imputation	VGAE	Unsupervised	Learns the intrinsic data distribution via masked gene expression reconstruction, addressing the 'over‐smoothing' issue in GCNs and preserving local details and heterogeneity.	Input: omics Data scale: 3460‐80 000 spots Evaluation metrics: ARI: 0.684 Pearson Correlation: +0.306
BLEEP^[^ ^179^ ^]^	Denoising and Imputation	CLIP (ResNet)	Self‐supervised	Relies solely on histological images for prediction, avoiding the curse of dimensionality and demonstrating robustness to experimental artifacts.	Input: omics and imaging Data scale: 9269 spots Evaluation metrics: R^2^: 0.217–0.173
stDCL^[^ ^180^ ^]^	Denoising and Imputation	GCN(CL)	Self‐supervised	Facilitates reconstruction of spatial hierarchies while strengthening layer‐specific gene expression signals.	Input: omics Data scale: 1200–30 000 cells Evaluation metrics: PCC: 0.502
stImpute^[^ ^181^ ^]^	Denoising and Imputation	AE, GraphASGE, ESM‐2	Self‐supervised	Incorporates functional relevance via ESM‐2‐based gene networks, enhancing interpretability beyond expression similarity.	Input: omics and imaging Data scale: 2000–1.3 M cells Evaluation metrics: MSE: 0.45–0.48 CSS: 0.66–0.74
STAMP^[^ ^188^ ^]^	Dimension Reduction	SGCN,Topic modeling	Unsupervised	Provides end‐to‐end interpretable dimension reduction with probabilistic representations, flexibly capturing cellular heterogeneity and scaling well across diverse spatial transcriptomics scenarios.	Input: omics Data scale: 39 220–93 206 spots Evaluation metrics: cLISI:0.96 KBET: 0.08
SpaHDmap^[^ ^189^ ^]^	Dimension Reduction	GCN, U‐net, NMF	Self‐supervised	Generates high‐resolution embeddings that reveal fine‐grained spatial structures, with multimodal processing capability and strong biological interpretability.	Input: omics and imaging Data scale: 167 780 cells Evaluation metrics: ARI: 0.81 MAE: 0.09
SPADE^[^ ^193^ ^]^	Identification of SVGs	VGG‐16	Self‐supervised	Through deep integration of ST data with histological images, SPADE identifies genes that are not only spatially variable but also closely associated with underlying tissue morphology.	Input: omics and imaging Data scale: 267–3813 spots Evaluation metrics: ARI: 0.324 Classification accuracy: 90.51%
GASTON^[^ ^194^ ^]^	Identification of SVGs	DNN	Self‐supervised	By simulating tissue slice topography, it captures both sharp, discontinuous gene expression changes at spatial domain boundaries and smooth expression gradients within domains, enhancing the biological relevance of SVG identification.	Input: omics Data scale: 3900–9985 spots Evaluation metrics: Spatial coherence score: 0.86 AUPRC: 0.31 ARI: 0.59 F‐measure: 0.74
PROST^[^ ^195^ ^]^	Identification of SVGs	GAT	Unsupervised	Introduces an interpretable quantitative metric (PI) for identifying and ranking SVGs, significantly enhancing spatial domain segmentation performance of PNN and other mainstream models such as STAGATE and SpaceFlow.	Input: omics Data scale: 19 109 spots Evaluation metrics: ARI: 0.474 NMI: 0.610 Moran's I: 0.384–0.122
GAADE^[^ ^196^ ^]^	Identification of SVGs	GAT	Unsupervised	identified SVGs exhibit clear spatial expression patterns, with flexible parameter settings that allow users to prioritize either spatial localization precision or detection quality based on research needs.	Input: omics Data scale: 2695–4788 spots Evaluation metrics: ARI: 0.60 Moran's I: 0.5428 Geary's C:0.5437
STAIG^[^ ^41^ ^]^	Spatial Clustering	GNN, BYOL	Self‐supervised	Utilizes image‐guided pre‐clustering to reduce false‐negative impact, and eliminates batch effects by learning local commonalities without requiring prior spatial alignment.	Input: omics and imaging Data scale: 2179–19 285 spots Evaluation metrics: ARI: 0.84 NMI: 0.78 SC: 0.40 DB: 0.87 BatchKL: 0.14 ILISI: 2.95
SpaGCN^[^ ^42^ ^]^	Spatial Clustering/Identification of SVGs	GCN	Unsupervised	As an early and innovative model, it successfully integrates ST data with histological images to jointly perform clustering and SVG identification.	Input: omics and imaging Data scale: 224–3353 spots Evaluation metrics: ARI:0.522 Moran's I:0.54
GraphST^[^ ^186^ ^]^	Spatial Clustering	GNN	Self‐supervised	Enhances spatial clustering and biological relevance by learning local microenvironments via contrastive learning, while integrating multi‐sample alignment and deconvolution in one framework.	Input: omics Data scale: 72–92 928 spots Evaluation metrics: ARI: 0.64 ILISI: 1.846
STAGATE^[^ ^203^ ^]^	Spatial Clustering	GATAE	Unsupervised	In low‐resolution settings, a cell type–aware module enables pre‐clustering to refine tissue boundary detection while simultaneously denoising and learning key spatial expression patterns.	Input: omics Data scale: 3498–50 000 spots Evaluation metrics: ARI: 0.60 NMI: 0.65
ResST^[^ ^204^ ^]^	Spatial Clustering	Residual graph learning,	Self‐supervised	Quantifies the impact of biological effects on clustering and employs domain adaptation based on Margin Disparity Discrepancy (MDD) theory with strict generalization bounds to achieve more accurate batch correction.	Input: omics and imaging Data scale: 3639–3844 spots Evaluation metrics: ARI: 0.792 SC: 0.161 DB: 1.676 CH: 284.062
DeepST^[^ ^206^ ^]^	Spatial Clustering	Inception v3, VGAE, DAN	Unsupervised	Enhances morphological feature extraction using a pre‐trained CNN and applies adversarial learning to effectively correct batch effects.	Input: omics and imaging Data scale: 3639–4000 spots Evaluation metrics: ARI: 0.798 SC: 0.421 DB: 1.258
SPACEL^[^ ^207^ ^]^	Spatial Clustering	VAE, GCN, Adversarial learning	Semi‐supervised	Provides a comprehensive ST data processing suite, including Spoint for deconvolution, Splane for spatial clustering across multiple sections, and Scube for 3D tissue reconstruction.	Input: omics Data scale: 3000–4000 spots Evaluation metrics: PCC: 0.73 SSIM: 0.69 RMSE: 0.05 JSD: 0.41 AS: 0.93
STMSGAL^[^ ^208^ ^]^	Spatial Clustering	GATE	Self‐supervised	Integrates multi‐level encoder features to capture comprehensive data structures, and employs a clustering‐guided self‐supervised module with pseudo‐labels for improved robustness.	Input: omics Data scale: 2264–5913 spots Evaluation metrics: ARI: 0.606 DB: 1.155 CH: 1010.724
MAFN^[^ ^209^ ^]^	Spatial Clustering	GCN	Unsupervised	Enhances feature discriminability via the CCR strategy and adaptively fuses multi‐source information through the CAM module, yielding more effective and robust representations for clustering.	Input: omics Data scale: 32 285–36 601 genes Evaluation metrics: ARI: 0.82 NMI:0.78
STAGUE^[^ ^210^ ^]^	Spatial Clustering	GCN	Unsupervised	Introduces a spatial learner to construct an additional view, enabling joint optimization of gene expression and spatial structure across three views for both spatial clustering and cell‐cell communication analysis.	Input: omics Data scale: 167–4788 spots Evaluation metrics: ARI: 0.841 AMI: 0.820
conST^[^ ^211^ ^]^	Spatial Clustering	GNN, MAE	Self‐supervised	Employs a multi‐level contrastive learning framework across data modalities and granularities, with GNNExplainer for interpretability, enhancing model credibility in biological applications.	Input: omics and imaging Data scale: 971–3278 spot Evaluation metrics: ARI: 0.65 SC: 0.8 CHS: 603 DBI: 1.8
stMVC^[^ ^212^ ^]^	Spatial Clustering	GATE, SimCLR	Semi‐supervised	Constructs two independent graph views—Histological Similarity Graph (HSG) and Spatial Location Graph (SLG)—and incorporates weak supervision from biological priors (e.g., annotated tumor regions) to guide embedding learning.	Input: omics and imaging Data scale: 3460–4789 spots Evaluation metrics: ASW: 0.44
SiGra^[^ ^213^ ^]^	Spatial Clustering	Transformer	Self‐supervised	Effectively integrates image and transcriptomic features through three parallel encoder–decoder branches, achieving clustering results (measured by ARI) closer to pathologist‐annotated gold standards than classical methods such as Seurat and BayesSpace.	Input: omics and imaging Scale: 3431–4221 spots Evaluation metrics: ARI: 0.62
SpaGT^[^ ^214^ ^]^	Spatial Clustering	Transformer	Unsupervised	Introduces structure‐reinforced self‐attention to iteratively refine graph structures, offering strong generalizability and stable performance on both high‐ and low‐resolution ST data without relying on additional modalities.	Input: omics and imaging Data scale: 1848–41 786 spots Evaluation metrics: ARI: 0.805 Moran's I: 0.664
FOCUS Framework^[^ ^216^ ^]^	Cell Type Annotation	GCN	Semi‐supervised	introduces a novel approach based on subcellular RNA spatial distribution, achieving high annotation accuracy and strong interpretability by quantifying gene importance and revealing pathways linked to cell identity, while maintaining high performance with limited labeled data.	Input: omics Data scale: 300 000–766 313 cells Evaluation metrics: F1: 0.909 Accuracy: 0.948
Spatial‐ID^[^ ^217^ ^]^	Cell Type Annotation	DNN, VGAE	Supervised	Demonstrates strong robustness to gene expression sparsity and is effectively applicable to 3D and large‐field (centimeter‐scale) tissue samples.	Input: omics Data scale: 31 299–80 186 cells Evaluation metrics: Accuracy: 92.75% Weighted F1: 0.9209
SPANN^[^ ^218^ ^]^	Cell Type Annotation	VAE	Supervised	achieves cell‐type‐level alignment through optimal transport, enables robust discovery of novel cell types with an expert ensemble system, and uniquely integrates spatial information via regularization techniques.	Input: omics Data scale: 4382–15 413 1549–3166 Evaluation metrics: ACC: 0.831 NMI: 0.772 ARI: 0.792
scBOL^[^ ^219^ ^]^	Cell Type Annotation	GCN	Semi‐supervised	Effectively addresses cross‐dataset cell type identification by employing bipartite prototype alignment, with strong capability in handling batch effects and discovering novel cell types.	Input: omics Data scale: 45 958–173 968 cells Evaluation metrics: Accuracy: 95.8%
STELLAR^[^ ^220^ ^]^	Cell Type Annotation	GCN	Semi‐supervised	The learned cell embeddings are applicable to both cell classification and the identification of higher‐order tissue structures, such as immune follicles, that extend beyond individual cellular neighborhoods.	Input: omics Data scale: 619 186–45 958 Evaluation metrics: Accuracy: 0.93 F1: 0.82
SpaDecon^[^ ^223^ ^]^	Deconvolution of Spatial Spots	SAE	Semi‐supervised	Integrates multimodal data to account for the tendency of spatially adjacent and histologically similar regions to share cell type compositions, while demonstrating high efficiency in speed and memory usage.	Input: omics and imaging Data scale: 74 973–100 064 cells 224–3798 spots Evaluation metrics: MSE: 0.004 JSD: 0.28
SD2^[^ ^224^ ^]^	Deconvolution of Spatial Spots	GCN, AE	Semi‐supervised	Treats high dropout rates as informative patterns rather than noise, and uses them to guide feature gene selection, representing a fundamental innovation at the feature selection level.	Input: omics Data scale: 1927–16 119 cells 428–3355 spots Evaluation metrics: RMSE: 0.06 JSD: 0.21 R: 0.57
STdGCN^[^ ^225^ ^]^	Deconvolution of Spatial Spots	GCN	Semi‐supervised	Employs a unique dual‐GCN parallel architecture and introduces an optimized pseudo‐ST point generation method to address the challenge of rare cell types.	Input: omics Data scale: 93 450–1.1 M cells 59–3115 spots Evaluation metrics: RMSE: 0.05 JSD: 0.002
SPADE^[^ ^226^ ^]^	Deconvolution of Spatial Spots	SpaGCN	Supervised	Uses a domain‐first strategy, achieving high true positive and low false positive rates in detecting correct cell types within each domain.	Input: omics and imaging Data scale: 47 209–22 000 cells 700–2000 spots Evaluation metrics: mAD: 0.007 RMSD: 0.015 R: 0.997
CLPLS^[^ ^227^ ^]^	Deconvolution of Spatial Spots	GCN, Contrastive learning	Self‐supervised	By integrating multi‐omics data, CLPLS resolves spatial cell type distribution and enables exploration of spatially epigenomic heterogeneity across tissues.	Input: omics Data scale: 4281–15 095 cells 490–53 208 spots Evaluation metrics: PCC: 0.92 SSIM: 0.91 RMSE: 0.12 JSD: 0.35 AUC: 0.99
SpatialcoGCN^[^ ^228^ ^]^	Deconvolution of Spatial Spots	VAE, GCN	Self‐supervised	In addition to deconvolution, introduces SpatialcoGCN‐Sim to generate simulated ST data with spatial information, closely matching real data in spatial expression correlation.	Input: omics Data scale: 1040–29 519 cells 953–2376 spots Evaluation metrics: ARS: 0.96 PCC: 0.88 SSIM: 0.82 COSSIM: 0.92 RMSE: 0.09 JSD: 0.49
LETSmix^[^ ^229^ ^]^	Deconvolution of Spatial Spots	DNN, Adversarial learning	Supervised	Incorporates four types of spatial information through the innovative LETS filter and employs Mixup‐enhanced domain adaptation to address platform effects and sample imbalance.	Input: omics and imaging Data scale: 1733–57 530 cells 224–10 000 spots Evaluation metrics: AUC: 0.94 ER: 0.78 JSD: 0.04 Moran's I: 0.28
STdeconvolve^[^ ^230^ ^]^	Deconvolution of Spatial Spots	Topic modeling	Unsupervised	As an unsupervised method, STdeconvolve is not limited by predefined reference cell types and can identify unique cell types or condition‐specific cell states with altered gene expression in ST samples.	Input: omics Data scale: 260–57 397 spots Evaluation metrics: RMSE: 0.05
STRIDE^[^ ^231^ ^]^	Deconvolution of Spatial Spots	Topic modeling	Unsupervised	Learns biologically meaningful and interpretable cell type features through topic modeling, and aligns sequential tissue sections to reconstruct 3D spatial architecture.	Input: omics Data scale: 33 043–611 034 cells 1000–11 626 spots Evaluation metrics: PCC: 0.84 RMSE: 0.013
SMART^[^ ^232^ ^]^	Deconvolution of Spatial Spots	Topic modeling	Semi‐supervised	Allows incorporation of covariates (e.g., disease status, sex, treatment group) into deconvolution to quantify condition‐specific changes in cell‐type expression profiles, requiring only a simple marker gene list and minimal reference data.	Input: omics Data scale: 50–2702 spots Evaluation metrics: RMSE: 0.0565 PCC: 0.955
Cellpose^[^ ^49^ ^]^	Cell Segmentation	U‐net	Supervised	Pre‐trained on high‐quality datasets to accurately segment diverse cell types; the novel gradient flow algorithm effectively addresses challenges like uneven fluorescence labeling and signal loss in nuclear regions.	Input: omics and imaging Data scale: 100–1139 images Evaluation metrics: AP: 0.93(IoU = 0.5)
Cellpose 2.0^[^ ^50^ ^]^	Cell Segmentation	U‐net	Supervised	Supports fine‐tuning with minimal labeled data to overcome general model limitations on unseen image types; introduces a model zoo and human‐in‐the‐loop framework for model selection and segmentation refinement.	Input: omics and imaging Data scale: 608–3188 images Evaluation Metric: Improved AP: 0.32
Cellpose3^[^ ^51^ ^]^	Cell Segmentation	U‐net	Supervised	Jointly trained on multiple degradation types—denoising, deblurring, and upsampling—enabling high‐quality image restoration without requiring users to specify degradation type or source, thus improving inputs for downstream segmentation.	Input: omics and imaging Data scale: 8402 images Evaluation metrics: Improved AP: 0.7
BIDCell^[^ ^52^ ^]^	Cell Segmentation	U‐net 3+	Self‐supervised	Implements self‐supervised learning to eliminate reliance on ground truth, with biologically‐informed loss functions that guide optimization based on cell shape, size, and other morphological features.	Input: omics and imaging Data scale: 4000 patches(40x40) Evaluation metrics: Pearson cor: 0.95
CelloType^[^ ^238^ ^]^	Cell Segmentation	Transformer, DINO	Supervised	Employs end‐to‐end multi‐task learning to jointly optimize segmentation and classification, enabling accurate identification of both cells and nuclei, as well as segmentation of non‐cellular structures with large size variability.	Input: omics and imaging Data scale: 59–28 images Evaluation metrics: AP: 0.93(IoU = 0.5)
SCS^[^ ^239^ ^]^	Cell Segmentation	Transformer	Supervised	Designed for high‐resolution ST data without requiring extensive manual annotation, it leverages automatically segmented nuclei from stained images as positive samples and incorporates neighboring gene expression profiles and spatial positions for training, aligning more closely with the intrinsic nature of spatial transcriptomics.	Input: omics and imaging Data scale: 570 k–42 M spots Evaluation metrics: IoU: 0.75 Pearson cor: 0.88
UCS^[^ ^240^ ^]^	Cell Segmentation	CNN	Supervised	Efficient and user‐friendly; the two‐step strategy achieves accurate cell boundaries highly consistent with H&E staining while maintaining high transcript coverage.	Input: omics and imaging Data scale: 107 829–165 752 cells Evaluation metrics: F1: 0.84
segger^[^ ^241^ ^]^	Cell Segmentation	Heterogeneous GCN	Supervised	Extends nucleus‐based segmentation to capture cytoplasmic signals while minimizing contamination, achieving a sensitivity–accuracy balance.	Input: omics and imaging Data scale: 180 k cells Evaluation metrics: PMP: 0.26 MECR: 0.015
JSTA^[^ ^242^ ^]^	Cell Segmentation	EM algorithm	Supervised	Jointly optimizes cell segmentation and cell type annotation through iterative EM algorithm, enabling high‐precision localization of cellular subtypes.	Input: omics and imaging Data scale: 83–142 cell types Evaluation metrics: Improved accuracy: 45%
FICTURE^[^ ^243^ ^]^	Cell Segmentation	LDA	Unsupervised	A segmentation‐Free method, instead of defining explicit cell boundaries, it infers spatial factors directly at submicron‐resolution pixel level, while remaining scalable to ultralarge datasets.	Input: omics and imaging Data scale: 6.8 M–700 M transcripts Evaluation metrics: Accuracy: 0.975
GeneSegNet^[^ ^244^ ^]^	Cell Segmentation	FCN	Supervised	Transforms discrete RNA spatial coordinates into continuous 2D probability maps, enabling effective integration with DAPI images; introduces a recursive training strategy with alternating optimization to enhance robustness and performance on noisy‐labeled datasets.	Input: omics and imaging Data scale: 28–59 images Evaluation metrics: Image IoU: 0.73 Gene IoU: 0.64

### 4.6 Emerging AI Advances in Spatial Transcriptomics

Early AI applications in ST are constrained by limited data and underdeveloped algorithms, resulting in task‐specific models with narrow generalizability.^[^ ^24^ ^]^ However, the advent of large‐scale datasets and advanced architectures propels FMs to the forefront of current research.^[^ ^146^, ^245^, ^246^, ^247^, ^248^, ^249^, ^250^, ^251^ ^]^ Trained on massive corpora, these models learn transferable representations that support diverse tasks with minimal fine‐tuning.^[^ ^141^ ^]^ Complementing this development, AI agents^[^ ^252^, ^253^, ^254^, ^255^, ^256^ ^]^ leverage LLMs to automate analytical workflows and enable natural language‐driven interaction, further lowering the barriers to adoption.^[^ ^257^ ^]^ Together, FMs and AI agents herald a new era in AI applications for ST, with the former providing the representational engine and the latter delivering the interactive interface

Recent progress in FMs centers on two complementary strategies: encoding spatial context directly from transcriptomic profiles, and aligning histology with expression through cross‐modal learning. The first strategy is exemplified by several recent advances. Nicheformer operationalizes the hypothesis that a cell's transcriptomic profile encodes its spatial environment, jointly training on scRNA‐seq and ST data to transfer spatial information into dissociated cells.^[^ ^146^ ^]^ CELLama takes a different approach by transforming top‐expressed genes and metadata into natural language—like sentences that incorporate neighboring cell types for ST data; these sentences are then used to fine‐tuned the all‐MiniLM‐L12‐v2 model for identifying spatially patterned subpopulations.^[^ ^247^ ^]^ Similarly, stFormer focuses on cell‐cell communication, extending the Transformer architecture with cross‐attention to integrate ligand gene information with expression data.^[^ ^245^ ^]^ HEIST further advances this direction by constructing hierarchical graphs at both the gene and cell levels, enabling bidirectional interactions that link gene programs to tissue‐level phenotypes.^[^ ^249^ ^]^ In contrast to this direct encoding, the second strategy, cross‐modal alignment, is pioneered by methods such as OmiCLIP, which employs two independent encoders—a Vision Transformer for tissue images and a causal masking Transformer for gene expression—aligned through contrastive learning following the CLIP paradigm.^[^ ^246^ ^]^ Similarly, ST‐Align refines biological resolution by converting whole‐slide images into point‐level gene features and domain‐level functional representations, enabling more precise spatial annotation than conventional CLIP‐based frameworks (**Table** **5**).^[^ ^248^ ^]^

**Table 5:** Overview of Foundation‐Scale AI models in spatial transcriptomics.

Tool	Model	Features	Provided Weights	Parameter size	Cell size
Nicheformer^[^ ^146^ ^]^	Transformer	Jointly pretrains on scRNA‑seq and ST to transfer spatial context into dissociated cells.	Provided	49.3 M	110 M
stFormer^[^ ^245^ ^]^	Transformer	Integrates ligand gene signals via biased cross‑attention to unify ST datasets.	Provided	71.4 M	4.1 M
OmiCLIP^[^ ^246^ ^]^	ViT + Transformer	Aligns histology and transcriptomics via CLIP‑style contrastive learning.	Provided	599 M	2.2 Pairs
CELLama^[^ ^247^ ^]^	Transformer	Encodes cells as natural‑language‑like sentences with metadata for universal embeddings.	Provided	33 M	–
ST‐Align^[^ ^248^ ^]^	LDDMM	Aligns ST datasets with nonlinear deformation handling for precise correspondence.	Not Provided	–	1.3 M Spots
HEIST^[^ ^249^ ^]^	Hierarchical Graph Transformer	Links gene programs to tissue phenotypes via bidirectional multi‑level graphs.	Not Provided	5.2 M	22.3 M
scGPT‐spatial^[^ ^250^ ^]^	Transformer	Groups local spatial patches; MoE handles heterogeneous sequencing technologies.	Provided	50 M	30 M
SToFM^[^ ^251^ ^]^	Transformer	Captures macro, micro, and gene‑scale features; pretrained on 88 million cells.	Provided	45 M	88 M

These foundational representations directly enable the rise of AI agents, which translate model capabilities into automated, human‐AI collaborative workflows. AutoBA exemplifies full automation, where users define tasks in a YAML file, and the system executes end‐to‐end analysis with error correction via its ACR module.^[^ ^256^ ^]^ In contrast, SpatialAgent^[^ ^253^ ^]^ and CellAgent^[^ ^254^ ^]^ emphasize human‐AI collaboration: SpatialAgent offers both automated and co‐pilot modes for interactive analysis, while CellAgent extends this idea through a multi‐agent design to coordinate task execution and refinement. For multimodal integration STAgent leverages a LLM to jointly process textual and visual inputs, enabling histological image interpretation and integrated report generation.^[^ ^252^ ^]^ Meanwhile, CompBioAgent demonstrates practical deployment through web‐based accessibility, translating natural language queries into structured commands for streamlined database access and visualization (**Table** **6**).^[^ ^255^ ^]^

**Table 6:** Emerging AI‐agent frameworks for spatial transcriptomics.

Tool	Computational Requirements	Usage	Online Service	Features
STAgent^[^ ^252^ ^]^	Medium	Local deployment/API	Not provided	Enables human‐AI collaboration and supports full spatial biology workflows, including experimental design, multimodal data analysis, and hypothesis generation.
SpatialAgent^[^ ^253^ ^]^	Usage under construction	Usage under construction	Not provided	Supports human‐AI collaboration and is capable of handling the entire spatial biology research workflow, from experimental design and multimodal data analysis to hypothesis generation.
CellAgent^[^ ^254^ ^]^	Low	Web mode	Provided	Employs multi‐agent collaborative decision‐making to simulate a "deep reasoning" process, enabling task decomposition, execution, and optimization in a closed‐loop manner. It also incorporates the sc‐Omni toolkit for efficient tool integration.
CompBioAgent^[^ ^255^ ^]^	Medium	Local deployment/API	Provided	Fully operated through natural language with zero programming requirements. Integrates tools such as Cellxgene VIP^[^ ^258^ ^]^ and CellDepot^[^ ^259^ ^]^ for querying and visualizing various diseases and cell types.
AutoBA^[^ ^256^ ^]^	Low (API)/ High (Local Deploy)	Local deployment/API	Not provided	Applicable to both spatial transcriptomics and multi‐omics; highly automated, user‐friendly, and compatible with emerging bioinformatics tools.

Despite these advances, performance gains remain difficult to attribute to specific architectural choices, dataset composition, or training strategies, underscoring the need for standardized benchmarks and unified public datasets.^[^ ^141^ ^]^ Additionally, scaling laws—while still driving improvements—face diminishing returns, making efficiency‐oriented scaling preferable to unbounded growth.^[^ ^260^ ^]^ For AI agents specifically, reducing manual labor and technical barriers requires AI agents to move beyond proof‐of‐concept toward robust, validated implementations that domain experts can trust without extensive computational expertise.^[^ ^261^ ^]^

## 5 Conclusion: Synthesizing the Impact of AI in Transcriptomics

**Figure 7:**
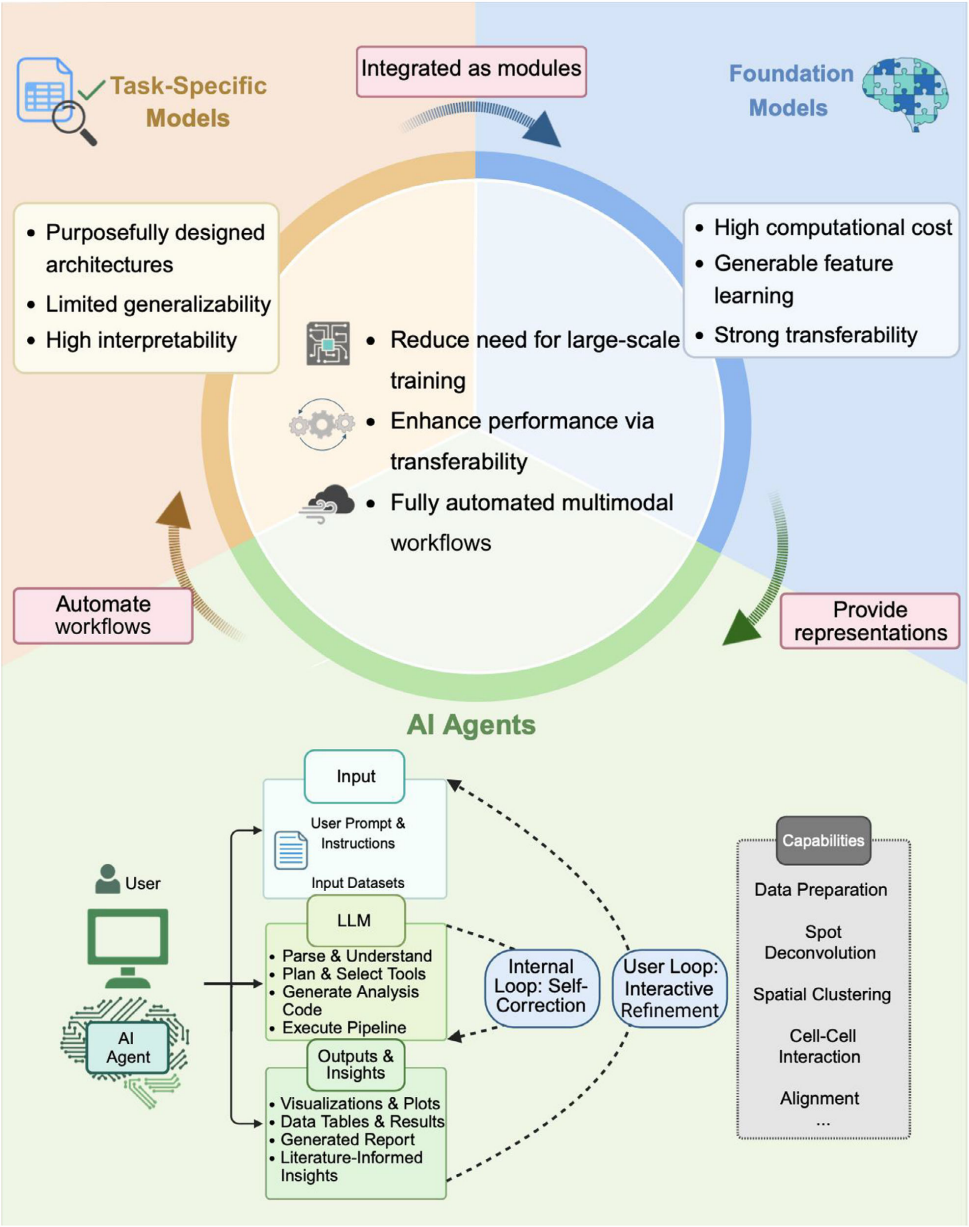
AI approaches can be broadly categorized into three groups: task‐specific models, which are interpretable but less generalizable; foundation models, which leverage large‐scale data to learn transferable representations; and AI agents, which integrate these modules to automate workflows and enable natural language–driven interaction.

The journey of single‐cell and spatial transcriptomics from nascent technologies to cornerstones of modern biology has been inextricably linked with and profoundly accelerated by advancements in AI. More than just providing incremental improvements, AI has fundamentally reshaped the analytical landscape by introducing a succession of increasingly powerful paradigms. The evolution of AI‐based methods can be understood through three interconnected stages: task‐specific models, FMs, and AI agents, which together form a dynamic and synergistic ecosystem (**Figure** **7**).

Initially, the field was dominated by task‐specific models, meticulously designed with specialized architectures to tackle discrete analytical challenges. Broadly applicable tools like scVI^[^ ^21^ ^]^ for data integration and SpaGCN^[^ ^42^ ^]^ for spatial domain identification exemplify this paradigm. They provided a robust and interpretable “computational toolbox” that not only solved critical problems like high dimensionality and noise but also established the analytical standards for the entire field. While limited in generalizability, their high interpretability made them indispensable for generating actionable biological hypotheses and building our foundational understanding of cellular heterogeneity.

The second paradigm emerges with the advent of FMs, representing a fundamental shift from bespoke engineering to universal biological knowledge. Pioneering models such as scGPT^[^ ^23^ ^]^ and GeneCompass^[^ ^34^ ^]^ epitomize this “pre‐train then fine‐tune” approach. By learning from hundreds of millions of cells, they have created transferable representations that democratize access to state‐of‐the‐art performance, with GeneCompass, for example, achieving a Macro F1 score of 0.98 and accuracy of 0.99 for cell type annotation in the human multiple sclerosis (hMS) dataset. This leap has unlocked unprecedented analytical power for complex tasks like perturbation prediction and cross‐species analysis, enabling atlas‐scale inquiry and setting the stage for standardized, reproducible big data science in biology.

Most recently, we are witnessing the rise of AI agents as the third and most interactive paradigm. Tools like SpatialAgent^[^ ^253^ ^]^ leverage the reasoning capabilities of LLMs to automate complex workflows and enable natural language–driven interaction. This transformative approach addresses the crucial need for accessibility, shifting the researcher's focus from the intricacies of coding and pipeline management to strategic scientific questioning and interpretation. It promises a future where sophisticated multi‐modal analyses can be orchestrated with minimal friction between hypothesis and result.

Crucially, these paradigms are not replacing one another but are becoming increasingly integrated. The true transformative power of AI in transcriptomics lies in its synergy. In this emerging ecosystem, task‐specific models provide the high‐fidelity, interpretable modules required for domain‐specific biological questions. ^2^FMs act as the powerful, generalizable engines that learn universal principles from vast data. AI agents function as the intelligent interface, orchestrating these specialized and generalized components to execute complex, multi‐step analyses.^[^ ^252^ ^]^

This multi‐layered ecosystem represents a fundamental change in how biological research is conducted. The convergence of interpretable, predictive, and autonomous capabilities is reshaping the very landscape of biological inquiry, moving the field toward a future where the cycle from hypothesis to insight is dramatically accelerated by a collaborative partnership between human researchers and AI.

## 6 Future Perspectives and Challenges

The synergistic ecosystem of task‐specific models, FMs, and AI agents points to a future in which artificial intelligence operates not merely as a tool but as a collaborative partner in scientific discovery. Realizing this vision requires tackling distinct challenges within each paradigm while forging deeper integrations across them.

Within the domain of task‐specific models, the priority must shift from developing standalone predictive tools to creating explainable (XAI), modular components that facilitate mechanistic biological discovery. The comparative advantage of these models lies not in computational scale but in interpretability—an essential quality for identifying actionable therapeutic targets and distinguishing correlation from causation. For example, InterPLM utilizes sparse autoencoders, commonly employed for dimensionality reduction, to perform interpretability analysis on the embeddings of protein language models.^[^ ^262^ ^]^ Such “white‐box” approaches are critical for verifying the outputs of larger, more opaque models, ensuring that downstream predictions—whether for target nomination or biomarker identification—are grounded in verifiable biological mechanisms rather than statistical artifacts.

In contrast, FMs face a complex situation involving computational accessibility and interpretability. FMs generally aim for versatility by training on massive curated datasets (e.g., > 110 million cells for CellFM^[^ ^35^ ^]^ and GeneCompass^[^ ^34^ ^]^) using high‐performance clusters. These models commonly scale from hundreds of millions to over a billion parameters (e.g., CellFM at 800 M, TranscriptFormer^[^ ^145^ ^]^ at 1.1B), entailing substantial hardware footprints (e.g., 1000 H100 GPUs for TranscriptFormer) and massive energy costs (e.g., 6912 for GeneCompass, 147 456 for CellFM). While such *de novo* training is prohibitive for most academic labs, a key benefit of FMs is the growing feasibility of fine‐tuning. Publicly available pretrained weights allow researchers to leverage these models on their own datasets; nevertheless, this still demands considerable resources, even for inference (e.g., UCE^[^ ^154^ ^]^ requires an 80GB GPU).

Given this substantial investment, a fundamental tension arises between predictive performance and biological interpretability. While models like OmiCLIP have advanced histology‐omics alignment by predicting molecular profiles from H&E images, extracting causal explanations linking morphology to gene expression remains challenging.^[^ ^246^ ^]^ Similarly, while attention mechanisms in FMs offer glimpses into gene regulation, they often capture correlational rather than causal associations,^[^ ^263^ ^]^ struggling to justify their resource consumption in tasks demanding rigorous biological reasoning, such as gene perturbation.^[^ ^23^, ^35^, ^144^ ^]^ In these contexts, FMs frequently fail to consistently outperform simple linear baselines, making their practical advantage difficult to discern.^[^ ^264^, ^265^, ^266^ ^]^ Consequently, researchers must prioritize flexible tool selection aligned with resources and needs, while recognizing that verifying the acquisition of generalizable biological rules requires more than just standard accuracy.^[^ ^266^ ^]^ This necessitates a shift toward rigorous benchmarking, as exemplified by the recent scDrugMAP, which achieved timely benchmarking of 10 FMs in the field of drug response.^[^ ^36^ ^]^ While our review synthesizes the architectural paradigms and theoretical potential of these models, scDrugMAP complements this by providing quantitative benchmarking of their specific utility in pharmacological contexts. By releasing tools that alleviate accessibility barriers, scDrugMAP and similar initiatives are essential for validating whether FMs can truly enhance translational pipelines compared to established methods.

Finally, compared to FMs, AI agents offer a different accessibility profile. While leveraging LLMs via APIs reduces infrastructure costs, local deployment for data privacy reintroduces high hardware requirements.^[^ ^267^ ^]^ Fundamentally, the efficacy of an agent relies on the underlying capabilities of the LLM, introducing the risk of hallucinations. Although multi‐agent frameworks attempt to mitigate this, they complicate the cost‐performance balance.^[^ ^267^, ^268^ ^]^ Furthermore, since LLMs currently function primarily as aggregators of existing human knowledge, their role is best suited for automating repetitive analytical tasks rather than generating serendipitous discovery.^[^ ^269^ ^]^ Therefore, the notion of fully autonomous analysis is premature. Future developments should prioritize “human‐in‐the‐loop” paradigms, where agents act as strategic assistants rather than replacements.^[^ ^270^ ^]^

These trajectories will converge on the vision of a “virtual laboratory” in the future.^[^ ^271^ ^]^ In this setting, AI agents will act as orchestrators, combining the predictive scale of FMs with the mechanistic insight of interpretable modules to design and analyze experiments in silico. The result will be not merely new tools, but a reshaped scientific workflow that accelerates the translation of computational predictions into biological understanding.

## Conflict of Interest

The authors declare no conflict of interest.

## Author Contributions

S.L., T.X., and Y.L. contributed equally to this work. C.Z. conceptualized and supervised the study. S.L. and T.X. drafted the manuscript with the help of C.W., Z.L., R.L., and Q.F. Y.L. generated all figures with the help of S.L. and T.X. All authors have read and approved the manuscript.
